# Gene expression modifications in Wharton’s Jelly mesenchymal stem cells promoted by prolonged in vitro culturing

**DOI:** 10.1186/1471-2164-14-635

**Published:** 2013-09-21

**Authors:** Valentina Gatta, Marco D’Aurora, Paola Lanuti, Laura Pierdomenico, Samantha Sperduti, Giandomenico Palka, Marco Gesi, Marco Marchisio, Sebastiano Miscia, Liborio Stuppia

**Affiliations:** 1 Department of Psychological, Humanities and Territory Sciences, School of Medicine and Health Sciences, University "G. d'Annunzio" Chieti-Pescara, via dei Vestini 31, 66013, Chieti, Italy; 2 Department of Neuroscience and Imaging, School of Medicine and Health Sciences, University "G. d'Annunzio" Chieti-Pescara, via dei Vestini 31, 66013, Chieti, Italy; 3 Department of Medicine and Aging Science, School of Medicine and Health Sciences, University "G. d'Annunzio" Chieti-Pescara, via dei Vestini 31, 66013, Chieti, Italy; 4 Department of Oral Health and Biotechnological Sciences, School of Medicine and Health Sciences, University "G. d'Annunzio" Chieti-Pescara, via dei Vestini 31, 66013, Chieti, Italy; 5 StemTeCh Group, via Polacchi 13, 66013, Chieti, Italy; 6 Aging Research Center, “Università G. d’Annunzio” Foundation, Via dei Vestini 31, 66013, Chieti, Italy; 7 Department of Translational Research and New Technologies in Medicine and Surgery, University of Pisa, via Risorgimento 36, 56126, Pisa, Italy

**Keywords:** Wharton’s Jelly, Mesenchymal stem cells, Microarray, Gene expression, In vitro expansion

## Abstract

**Background:**

It has been demonstrated that the umbilical cord matrix, represented by the Wharton’s Jelly (WJ), contains a great number of mesenchymal stem cells (MSCs), characterized by the expression of specific MSCs markers, shared by both human and animal models. The easy access to massive WJ amount makes it an attractive source of MSCs for cell-based therapies. However, as in other stem cell models, a deeper investigation of WJ-derived MSCs (WJ-MSCs) biological properties, probably modulated by their prolonged expansion and fast growth abilities, is required before their use in clinical settings. In this context, in order to analyze specific gene expression modifications occurring in WJ-MSCs, along with their culture prolongation, we investigated the transcriptomic profiles of WJ-MSCs after 4 and 12 passages of *in vitro* expansion by microarray analysis.

**Results:**

Hierarchical clustering analysis of the data set originated from a total of 6 experiments revealed that in vitro expansion of WJ-MSCs up to 12 passages promote selective over-expression of 157 genes and down-regulation of 440 genes compared to the 4^t^h passage. IPA software analysis of the biological functions related to the identified sets of genes disclosed several transcripts related to inflammatory and cell stress response, cell proliferation and maturation, and apoptosis.

**Conclusions:**

Taken together, these modifications may lead to an impairment of both cell expansion ability and resistance to apoptosis, two hallmarks of aging cells. In conclusion, results provided by the present study suggest the need to develop novel culture protocols able to preserve stem cell plasticity.

## Background

Mesenchymal stem cells (MSCs) are generally considered the main toolbox for cell-based therapies. Compared to embryonic stem cells (ESCs), MSCs exhibit the following advantages: accessibility with fewer ethical controversies [1], no reports of teratoma formation after transplantation, and versatile therapeutic applications [2-7]. Based on literature data, the phenotype of MSCs obtained from different sources is typically characterized by the expression of CD44, CD73, CD90 and CD105, representing the best suited markers currently used to characterize these cells, together with the lack of the expression of endothelial/hematopoietic markers (CD144, CD34, CD45) [8,9]. The umbilical cord (UC) is an extra-embryonic formation essential to provide feeding to the fetus during the intrauterine development. It has been shown that UC matrix, represented by the Wharton’s Jelly (WJ), surrounding umbilical vessels, contains a great number of mesenchymal cells, which have been characterized as expressing aforementioned markers, shared by MSCs in both human and animal models [10-12]. The abundant amount of WJ makes it an attractive source of MSCs for cell-based therapies [13,14]. However, as in other stem cell models, a deep investigation of WJ-MSCs biological properties is required before their use in clinical settings. In this context, a critical point is represented by the analysis of functional modifications affecting WJ-MSCs along with their prolonged *in vitro* cultures. In fact, a recent study highlighted the changes in protein expression profiling, along with the *in vitro* expansion of WJ-MSCs, probably related to the gradual impairment of their stem cell plasticity and of the biological mechanisms occurring in cellular aging [15]. In order to provide a different investigation model of the biological modifications occurring during WJ-MSCs *in vitro* growth, we analyzed the transcriptomic profile of the aforementioned cells following prolonged culture times [12^th^ passage compared to an early (4^th^) passage] by microarray analysis. The aim of the present study was to identify possible novel markers related to their in vitro prolonged expansion and to their fast growth abilities.

## Methods

### Cell isolation and culture

Institutional review board approval was obtained for all cell culture procedures. Fresh human UC (N = 5) were obtained from full-term births, after written informed consent was obtained from parents. UC were aseptically stored in sterile saline solution and processed within 6 hours from the partum to obtain WJ-MSCs, as previously described [15]. Briefly, after the removal of blood vessels, the extracellular matrix of WJ was scraped off, treated with 2 mg/ml collagenase IV (Sigma) for 16 hours at 37°C and then with 2.5% trypsin for 30 minutes at 37°C, under agitation. Finally, the obtained cell suspension was seeded in complete Human mesenchymal stem cell growth medium (hMSCGM, Lonza) and cultured in 5% CO_2_ in a 37°C incubator. When 80% of confluence was reached, the adherent fraction of cells was detached with 0.05% trypsin-EDTA, counted by Trypan Blue exclusion test, and reseeded at 3000 cells/cm^2^ to reach the 90% of confluence after 3–4 population doublings.

### Immunophenotype

WJ-MSCs were harvested at two experimental time points (4^th^ and 12^th^ culture passages) and were immediately incubated with 1 μg/10^6^ cells of fluorescein isotiocynate (FITC)-conjugated or phycoerythryne (PE)-conjugated antibody for 40 minutes at 4°C in the dark. Anti-CD73, anti-CD13, anti-CD90, anti-CD117, anti-CD14, anti-CD34, anti-CD105 and anti-CD45 (Becton Dickinson, San Jose, CA, USA), anti-CD29, anti-CD44 and anti-CD166 (Ancell, Bayport, MN, USA) antibodies were used. After a washing step, 10,000 events/sample were acquired on a FACSCalibur flow cytometer (two-lasers, four-color configuration) with CellQuest 3.2.1.f1 (BD) software; data were analysed using FlowJo™ software (TreeStar, Ashland, OR) [16].

### Doubling time and cell cycle analyses by bromodeoxyuridine incorporation assay

Exponentially growing WJ-MSCs were exposed to 10 μM bromodeoxyuridine (BrdU) (Sigma, St. Louis, MO, USA) for 1 h, then fixed in 70% ethanol and kept at 4°C before labeling as previously described [17]. To detect BrdU incorporation, cells were washed with PBS and treated with 1 ml of a solution containing 2 N HCl/0.5% Triton X-100 (Sigma) for 30 min at room temperature. 1 ml *per* sample of 0.1 M Na_2_B_4_O_7_ (pH 8.57) was added to stop the HCl reaction. Cells were then washed with 1 ml of a solution containing 0.5% Triton X-100/1% BSA, followed by an incubation for 30 min at room temperature in the dark with fluorescein isothiocyanate (FITC)-conjugated anti-BrdU antibody (BD Biosciences, San Jose, CA; dilution: 1:5 in 0.5% v/v Triton X-100). Cells were washed and resuspended in a solution containing 5 μg/ml Propidium Iodide (PI, Sigma) and 200 μg/ml RNase (Sigma). After 30 min of incubation biparametric BrdU/DNA data were acquired on a FACSCalibur flow cytometer (two-lasers, four-color configuration) with CellQuest 3.2.1.f1 (BD) software; data were analysed using FlowJo™ software (TreeStar, Ashland, OR) or ModFit LT™ software (Verity Software House, Toshan, ME, USA). Debris was excluded from the analysis by gating a forward scatter versus side scatter plot. Cell aggregates were excluded by gating FL2 area versus FL2 width [17].

### Telomere length assay

Genomic DNA was extracted from WJ-MSC at different passages using Wizard Genomic DNA Purification Kit (Promega) following the manufacturer's instructions. The length of telomere regions was assessed using the Telo TAGGG kit (Roche) according to the manufacturer's instructions. Appropriate controls, represented by DNA extracted from cells with long or short telomere regions, were also provided with the kit [15].

### Determination of cell senescence

The amount of senescent cells was evaluated in the different reported conditions by using the Senescence β-Galactosidase Staining Kit (Abcam, Cambridge, UK) in accordance to the manufacturer's instructions, as previously described [15].

### Adipogenic differentiation

To induce adipocyte differentiation, 10 × 10^3^ cells/cm^2^ were cultured in DMEM high glucose (HG) (Sigma) supplemented with 10% FBS (Gibco), 0.5 mM isobutyl-methylxantine (Sigma), 200 μM indomethacin (Sigma), 1 μM dexamethasone (Sigma) and 10 μg/ml insulin (Sigma). Cells were cultured, replacing the medium every 2–3 days. After 2–3 weeks of culture, cells contained neutral lipids in fat vacuoles; they were fixed in 10% formalin and stained with fresh oil red-O solution (Sigma) [15].

### Osteogenic differentiation

To induce osteogenic differentiation, 3 × 10^3^ cells/cm^2^ were cultured in MEM (Sigma) supplemented with 10% FBS (Gibco), 10 mMβ-glycerophosphate (Sigma), 0.2 mM ascorbic acid (Sigma), and 10 nM dexamethasone (Sigma), and cultured for 3–4 weeks, replacing the medium every 2–3 days. To demonstrate osteogenic differentiation, cultures were fixed and induced to the alkaline phosphatase reaction [15].

### Expression profiling

Total RNA was extracted from about 10^6^ cells/sample of two different WJ-MSCs cultures after 4 and 12 passages during their *in vitro* expansion, using the SVtotal RNA Izolation System kit (Promega, Madison, WI, USA). The purity and quantity of RNA was assessed using the Agilent 8453 Spectrophotometer (Agilent, Santa Clara, CA, USA). RNA quality was determined by both the evaluation of the rRNA band integrity, using agarose electrophoresis, and absorption readings at 260 and 280 nm. Extracted RNA was linearly amplified using the Amino AllylMessageAmp™ II aRNA Amplification Kit (Ambion, Austin, TX, USA). Five to ten μg of amplified aRNA were fluorescently labeled with Cy3-Cy5 cyanins and then hybridized on high-density array Human Whole Genome OneArray™ Microarray V5 (30,968 total probe; Biosense, Italy). Amplified aRNAs were used for microarray experiments carried out by hybridization of WJ-MSCs after 4 passages, compared to WJ-MSCs after 12 passages, for a total of 6 experiments (Table 1). The same biological samples have been compared at 4th and 12th passage. After hybridization, Cy3-Cy5 fluorescent signals were captured by a Confocal Laser Scanner "ScanArray Express" (Packard BioScience) and analyzed using the software "ScanArray Express-MicroArray Analysis System" version 3.0 (Perkin Elmer). Raw data of the performed experiments were recorded in the GEO public database (accession number: GSE34929). The values of the median signal intensity from each spot were subtracted from the local median background intensity. For each slide, after local background subtraction, a LOWESS algorithm was used for row data normalization, to evaluate signal to noise ratio and generate log ratios of sample *vs* reference signal. A gene was considered to be differentially expressed when showing an absolute value of log-ratio higher or equal to ± 0.5, an index that translates to a fold-change of 1.4 in transcript quantity. Analysis of data obtained by microarray experiments was carried out by means of hierarchical gene clustering [18] using Cluster 3.0 (open source 2006) and TreeView (Stanford University Labs) software. In order to include in clustering analysis only well measured transcripts, we selected spots with a present call (identified transcripts with measurable expression) in at least 80% of experiments and being > 1.7 fold up- or down regulated in at least 5 experiments. Identified clusters were then analyzed by the Ingenuity Pathways Analysis (IPA) software (Ingenuity Systems, Redwood City, CA), in order to classify genes based on their biological functions and disclose functional networks connecting specific genes. IPA infers and ranks networks by a score, expressed as a numerical value, which is a probabilistic fit between the amount of focus genes that are potentially eligible for a network composition and present on a given gene list, the size of the network, as well as all the molecules present in the Ingenuity Knowledge Base that can be part of such a network. To validate the microarray results, qRT-PCR analysis was performed on three down-regulated (p53, HSPE1 and HIST1H3C) and three up-regulated genes (IL1B, CREBBP and LYN) as evidenced by microarray experiments, using the housekeeping gene GAPDH as internal control to normalize the relative expressions of target genes. The quantitative RT-PCR was carried out in a total volume of 50 μl containing 1× TaqMan Universal PCR Master mix, no AmpErase UNG and 2 μl of cDNA using the TaqMan assay on the Abi 7900HT Sequencing Detection System. Genes primers and probe sets used were NM_000546 (p53), NM_002157 (HSPE1), NM_003531 (HIST1H3C), NM_004380 (CREBBP), NM_000877 (IL1B), NM_002350 (LYN), NM_002046 (GAPDH) (Integrated DNA Technologies, Coralville, Iowa, USA). The real time amplifications included 10 minutes at 95°C, followed by 48 cycles of 15 seconds at 95°C and 1 minute at 60°C. Relative expression levels were calculated for each sample after normalization against the housekeeping gene GAPDH, using the ΔΔCt method for comparing relative fold expression differences [19].

**Table 1:** **Phenotype and markers expression levels in WJ-MSC at 4**^**th **^**and 12**^**th **^**passage**

**Antigens**	**Phenotype**	**4**^**th **^**passage**	**12**^**th **^**passage**	**p <**
		**MFI ratio ± S.D.**		
**CD13**	+	11.6 ± 0.8	7.7 ± 0.8	**0.002**
**CD14**	-	1.3 ± 0.1	1.1 ± 0.1	0.222
**CD29**	+++	154.6 ± 7.3	141.9 ± 2.4	0.036
**CD34**	-	1.3 ± 0.1	1.1 ± 0.1	0.210
**CD44**	+++	173.8 ± 20.1	82.0 ± 8.7	**0.009**
**CD45**	-	1.1 ± 0.2	1.2 ± 0.2	0.308
**CD73**	++	49.5 ± 2.7	19.9 ± 1.1	**0.001**
**CD90**	++	78.4 ± 2.6	81.0 ± 3.2	0.226
**CD105**	+	8.7 ± 0.7	7.9 ± 0.5	0.218
**CD117**	-	1.8 ± 0.2	1.5 ± 0.2	0.152
**CD133**	-	1.5 ± 0.1	1.2 ± 0.1	0.094
**CD144**	-	1.2 ± 0.1	1.3 ± 0.2	0.320
**CD146**	+	10.5 ± 0.6	11.3 ± 0.6	0.230
**CD166**	+	14.4 ± 0.9	6.8 ± 1.2	0.014
**CD326**	-	1. 1 ± 0.1	1.1 ± 0.1	0.420
**HLA-ABC**	+	24.0 ± 0.4	24.9 ± 0.6	0.023
**HLA-DR**	-	1.3 ±0.1	1.1 ± 0.1	0.341

– negative expression; + moderate expression; ++ positive; +++ high expression; MFI Ratio is the average of five different biological samples ± standard deviation; Bold values represent MFI Ratio With p < 0.01; Cut-off positivity MFI Ratio > 2.

## Results

### Cells isolation, culturing and characterization

**Figure 1:**
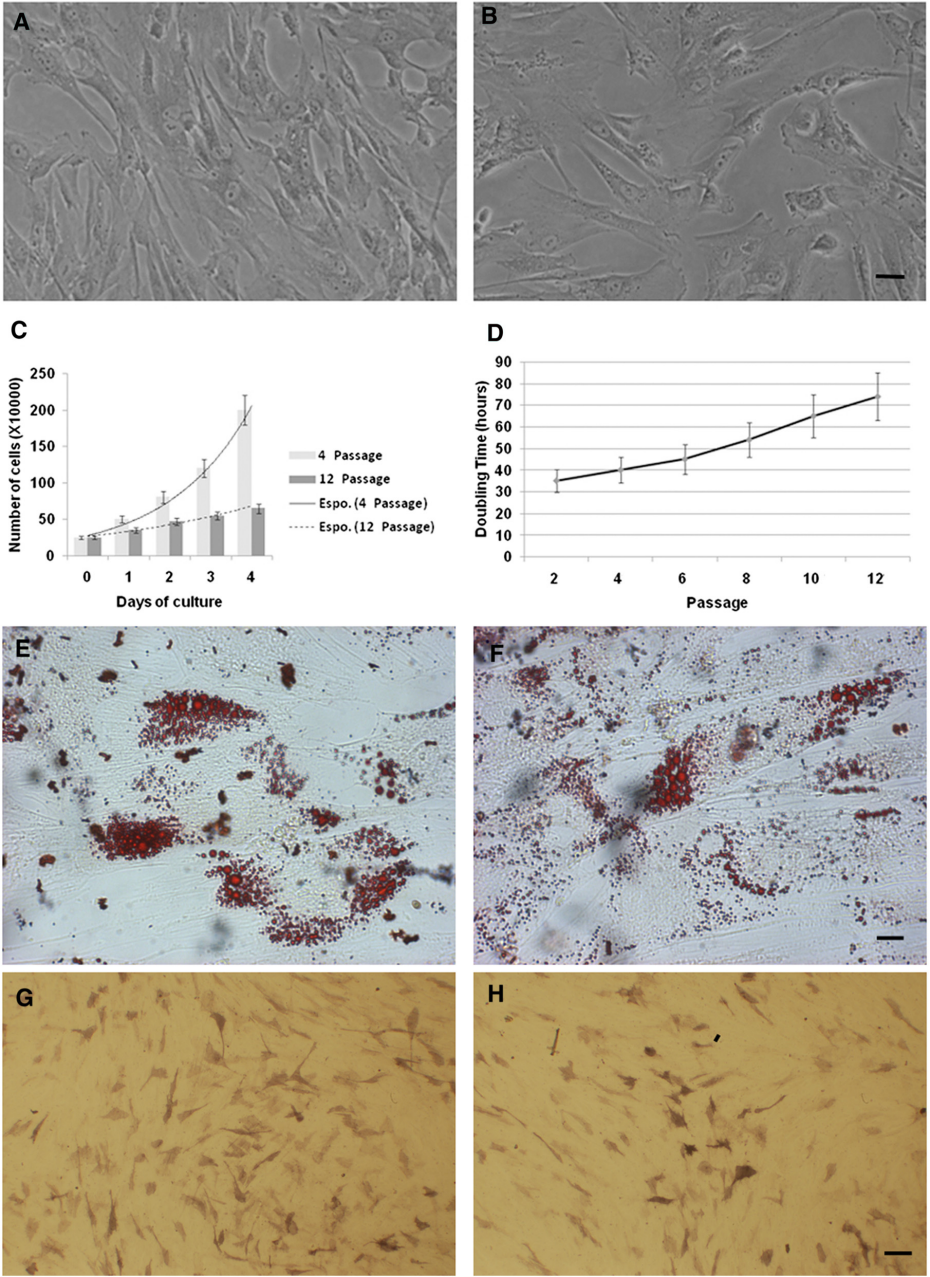
**WJCs characterization.** Light microscopic micrographs of WJ-MSCs in monolayer at the 4^th^**(A)** and the 12^th^**(B)** culture passages. In monolayer culture, cells assumed a polymorphic, fibroblast-like morphology, which was maintained throughout the time of culture (scale bar 40 μm). Growth characterization of WJ-MSCs during the *in vitro* expansion (4^th^ and 12^th^ passage) measured by cell count **(C)** and by the BrdU incorporation assay, allowing the doubling time evaluation **(D)**. The ability of WJ-MSCs to differentiate into the adipogenic lineage at the 4^th^**(E)** and the 12^th^**(F)** culture passages was evidenced by the intracellular accumulation of neutral lipid vacuoles (red oil staining) (scale bar 20 μm). Osteogenic differentiation at the 4^th^**(G)** and the 12^th^**(H)** culture passages was indicated by the increase in alkaline phosphatase reaction (scale bar 80 μm). Light microscopic micrographs A, B, E, F, G and H are representative of five separate biological samples.

Cells isolated from the human WJ displayed a consistent spindle-shaped elongated fibroblast-like morphology at the 4^th^ passage, a feature retained up to the 12^th^ passage (Figure 1A, B). A representative immunophenotype of cells used in our experiments is reported in Additional file 1: Figure S1. A positive response pattern expression of CD13, CD29, CD44, CD73, CD90, CD105, CD146, CD166, HLA-ABC markers and a negative reactivity for CD14, CD34, CD45, CD117, CD133, CD144, CD326, HLA-DR were detected at both 4^th^ and 12^th^ passages (Table 1). Such a high homogeneous marker expression suggests that non-stem cell populations did not significantly contaminate samples used in the present study. On the other hand, the expression of CD13, CD44 and CD73 underwent a progressive and statistically significant reduction at the 12^th^ passage (Table 1) while the variation of the adhesion molecule expression (CD14, CD44 and CD73) is in accordance with a stem cell aging process, during *in vitro* expansion. The steady ability of WJ-MSCs to differentiate into both adipogenic and osteogenic lineages at the 4^th^ and the 12^th^ passage was evidenced (Figure 1). After their expansion, cells showed a homogeneous diploid content during the G1 cell-cycle phase (Additional file 2: Figure S2). Furthermore, G1 and G2 cell-cycle checkpoints appeared intact. This finding is consistent with actively cycling cells. The long telomeric end of DNA extracted from WJ-MSCs at all examined passages also confirms that these cells preserve their capability to undergo a high number of cellular divisions up to the 12^th^ passage (data not shown) [15]. Accordingly, a low frequency of cells staining positive for β-galactosidase was found at both studied passages (4^th^ and 12^th^). On the other hand, long-term *in vitro* culture passages led to an impairment of cell expansion ability, as demonstrated by different exponential curves of growth at the 4^th^ and the 12^th^ passage, as demonstrated by cell counts (Figure 1C). These data were confirmed by the analysis of the doubling time obtained through the BrdU incorporation assay, associated to the DNA staining (Figure 1D): results evidenced that the doubling time progressively increased from 32 h at the 4^th^ passage to 74 h at the 12^th^ passage (p < 0.001).

### Gene expression profile of WJ-MSCs at the 4^th^ passage vs the 12^th^ passage

**Figure 2:**
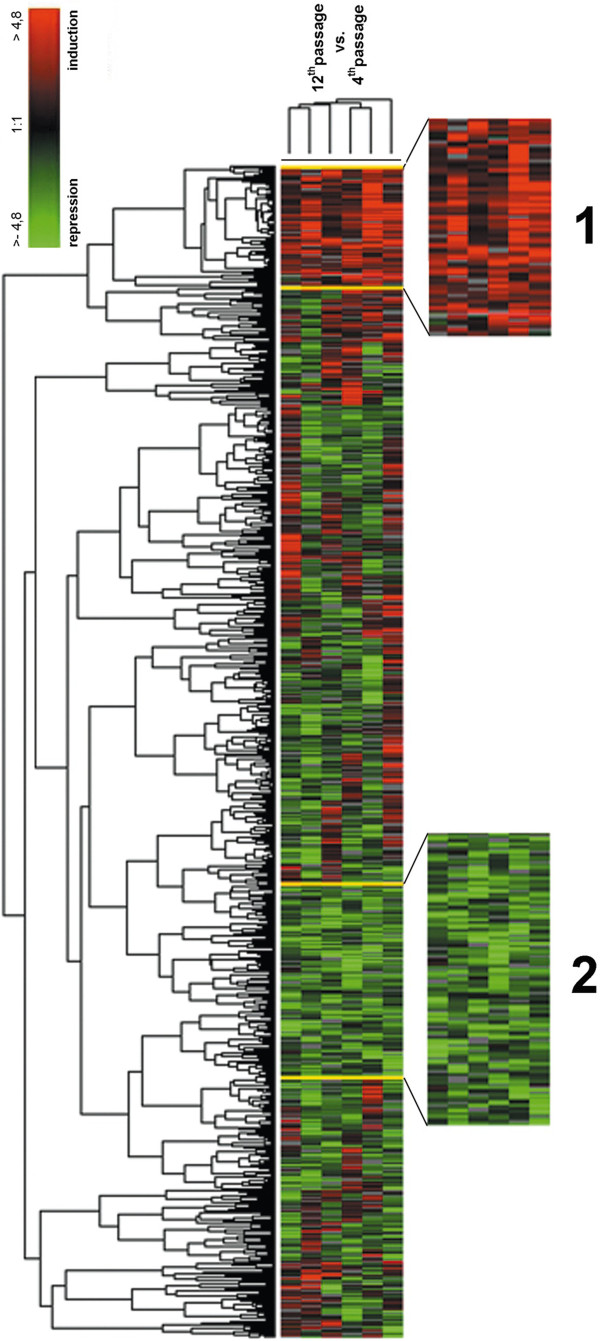
**Hierarchical clustering analysis.** The cluster analysis shows the presence of two different clusters composed respectively by 157 up-regulated transcripts (cluster 1) and 440 down-regulated genes (cluster 2). In the figure, all the relevant genes are grouped according to their expression values, shown as log ratios. Each row corresponds to one gene, each column to the different 6 microarray experiments. The quantitative changes in gene expression across all the samples are represented in different colors: red indicates over-expressed genes, and green indicates down-regulated genes. Black bars indicate no changes in gene expression. Missing data points are represented as gray bars. The top labels indicate the different experiments.

**Figure 3:**
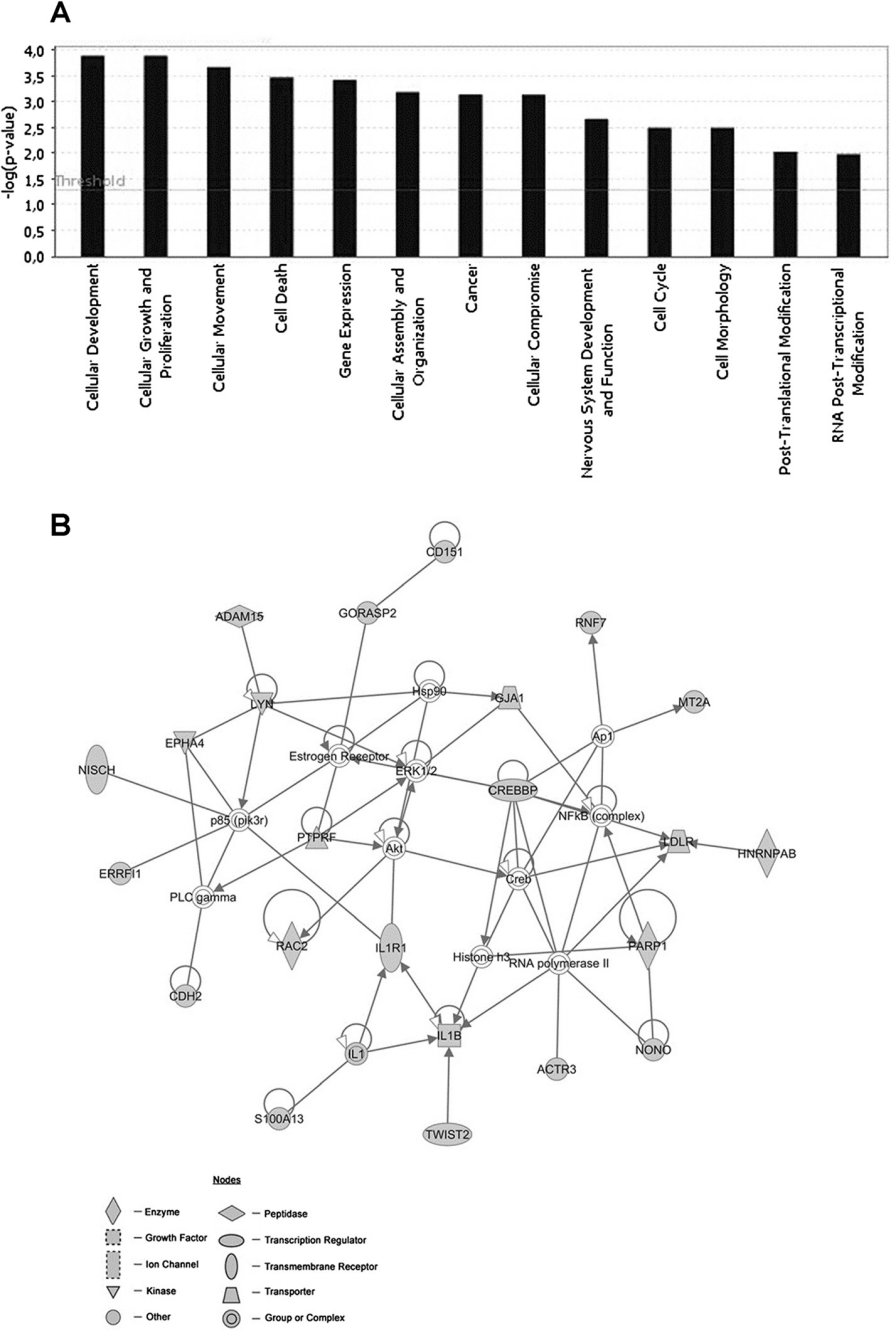
**IPA functional analysis and first top network associated with the Cluster 1. (A)** Bar chart shows key function associated with genes found to be up-regulated (cluster 1) in WJ-MSCs after 12 passages of their *in vitro* expansion, as compared to WJ-MSCs at the 4^th^ passage. **(B)** Network cluster 1: in grey are represented the genes up-regulated in WJ-MSCs after 12 passages of their *in vitro* expansion, as compared to WJ-MSCs at the 4^th^ passage. Transcripts not modulated along with the different passages are represented in white. Arrows indicate that a molecule acts on another molecule, while lines indicate a bind between two molecules.

**Figure 4:**
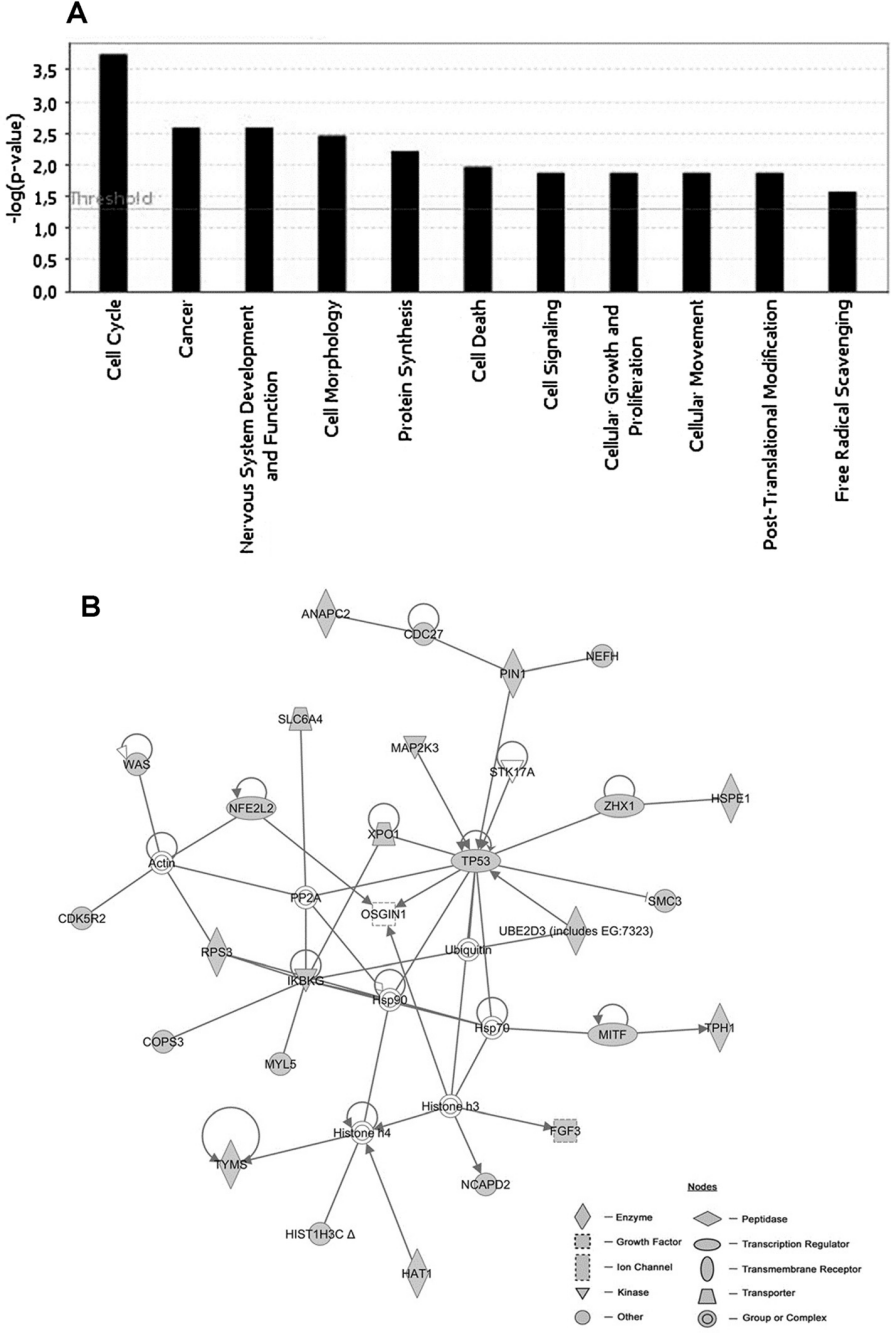
**IPA functional analysis and first top network associated with the Cluster 2. (A)** Bar chart shows key function associated with genes found to be down-regulated (cluster 2) at the 12^th^ passage of WJ-MSCs as compared to cells at the 4^th^ passage. **(B)** Network cluster 2: in gray are represented the down-regulated genes at the 12^th^ passage of WJ-MSCs *in vitro* expanded, as compared to the same cells at the 4^th^ passage. Transcripts not modulated along with the different passages are represented in white. Arrows indicate that a molecule acts on another molecule, while lines indicate a bind between two molecules.

To gain insights on the global changes in gene expression of human WJ-MSCs, produced by 12 passages *in vitro* expansion as compared to 4 passages, we performed a hierarchical clustering analysis of the data set originated from a total of 6 experiments (3 biological and 3 technical replicates). On a total of 30,968 transcripts investigated by the array, the analysis revealed that 12 passages in vitro expansion of WJ-MSCs promote the selective over-expression of 157 genes (cluster 1), while 440 genes were down-regulated (cluster 2) as compared to 4 passages expansion cells (Figure 2; Additional file 3: Table S1; Additional file 4: Table S2). Ingenuity Pathway Analysis (IPA) was carried out to investigate the main functions played by the selected clusters of genes. The up-regulated gene dataset (n = 157) was mainly composed by genes involved in functions regarding cellular development, cellular growth and proliferation, cellular movement, cell death, cellular assembly and organization, gene expression, cancer, cellular compromise, nervous system development and function, cell cycle, cell morphology, post-translational modifications and RNA post-transcriptional modification (Figure 3A). This analysis also demonstrated that down-regulated genes (n = 440) were involved in functions regarding cell cycle, cancer, nervous system development and function, cell morphology, protein synthesis, cell death, cell signaling, cellular growth and proliferation, cellular movement, post-translational modification and free radical scavenging (Figure 4A).

### Networks associated with transcripts selectively modified at the 12^th^ passage in vitro expansion

IPA software predicts functional networks based on known protein-protein and functional interactions. We therefore employed IPA to study how the genes selectively changing their expression at the 12^th^ passage as compared to the 4^th^ passage were interacting in specific networks.

IPA analysis of the up-regulated genes indicated 8 networks with a score ranging from 46 to 16. The first top network generated by IPA (score = 46) (Figure 3B) is composed by: i) genes around the gene node IL1, namely IL1B, IL1R1, S100113 and TWIST2, that participate in the inflammatory response and apoptosis; ii) genes around AKT gene node, namely RAC2 and PTPRF, involved in protein phosphorylation, cell differentiation, proliferation and maturation; iii) other genes not linked to any specific node, whose function is related to the transcription regulation (PARP1, HNRNPAB, NONO CREBBP), cytoskeletal organization (ACTR3, NISCH), MSC differentiation (EPHA4, MT2A, CDH2, RAC), cell stress response (ERRFI1), inhibition of apoptosis (RNF7, TWIST2, IL1B), cell proliferation and maturation (LYN) (Table 2). IPA analysis of the down-regulated genes showed 14 networks with a score ranging from 45 to 11. The first top network generated by IPA (score = 45) (Figure 4B) is composed by: i) genes around p53 gene node, namely UBE2D3, MAP2K3, ZHX1, XPO1, PIN1, CDC27, ANAPC2; ii) other genes not linked to any specific node, whose function was related to cell proliferation and pluripotency (TYMS, FGFR3, MITF and NCAPD2), oxidative stress (RPS3, NFE2L2, HSP1, HAT1), NF-κB signaling (IKBKG, MYL5), apoptosis (SLC6A4, TPH1) and histone modification (HAT1, HIST1H3C) (Table 3).

**Table 2:** **Up-regulated gene functions in cell cultures at 12**^**th **^**passage as compared to cells at 4**^**th **^**passage**

**Class**	**Gene**	**Description**	**Functions**	**Ref**
IL1 NODE	IL1	Pro-inflammatory cytokine involved in host defense	Activation of NF-kappaB; Induction of acute and chronic inflammation	[20]
	IL1B	Cytokine activated by Caspase 1	Cell proliferation; Cell differentiation; Apoptosis	[21]
	IL1R1	Cytokine receptor 1	Immune and inflammatory response	[20]
	S100A13	Calcium binding protein induced by inflammatory stress	Cell cycle progression; Cellular differentiation	[22]
	TWIST2	MSCs marker	Negative regulator of IL1B; Cell lineage determination; Cellular differentiation	[23,24]
AKT NODE	AKT	Protein kinase B	Cellular growth; Mitosis; Cellular differentiation	[25]
	RAC2	Member of Rho GTPase family	Proliferation; Cellular differentiation; Cytoskeletal organization; Cellular adhesion; Membrane trafficking, Transcriptional regulation	[26,27]
	PTPRF	LAR protein tyrosine phosphatase sigma family	Neuronal differentiation; Cellular development	[28]
Transcription regulators	CREBBP	CREB binding protein	Histone acetyltransferase activity; Transcriptional regulation	[29]
	PARP1	Poly (ADP-ribose) polymerase	Cellular proliferation; Cellular differentiation	[30]
	HNRNPAB	Ribonucleoprotein associated with pre-mRNAs	RNA processing and trafficking; Cellular differentiation	[31]
	NONO	RNA-binding protein	Transcriptional regulation; RNA splicing; RNA retention in nucleus	[32]
Cytoskeleton organization	ACTR3	Actin-related protein	Neuronal differentiation	[33]
	NISCH	Nischarin	Negative role in cell migration	[34]
Neural markers	EPHA4	Ephrin recepotor of protein-tyrosine kinase family	Nervous system development	[35]
	MT2A	Melatonin recepror 2	Expressed in neural progenitor	[36]
	CDH2	Cadherin	Cellular differentiation via RAC	[37]
	ERRFI1	Cytoplasmic protein	Induced by cellular stress; Cell signalling	[38]
	RNF7	Ring finger protein	Part of protein degradation machinery; Antiapoptotic activity via JUN	[39]
	LYN	v-yes-1 Yamaguchi sarcoma viral related oncogene	Tyrosine kinase activity; Cellular proliferation	[40]

**Table 3:** **Down-regulated gene functions in cell cultures at 12**^**th **^**passage as compared to cells at 4**^**th **^**passage**

**Class**	**Gene**	**Description**	**Functions**	**Ref**
TP53 NODE	TP53	Major tumor protein suppressor	Cell cycle; Apoptosis; Senescence	[41,42]
	UBE2D3	Member of E2 ubiquitin conjugating enzyme family	Protein degradation machinery	[43]
	MAP2K3	Protein kinase activated by environmental stress	Gene expression regulation; Mitosis; Cellular differentiation; Cellular proliferation; Apoptosis; Senescence	[44]
	ZHX1	Zing finger and homeoboxes gene family	Maintenance of TP53 gene silencing	[45]
	XPO1	Exportin 1	Protein trafficking; Localization of cyclin b; Localization of MPAK; Nuclear export of TP53	[46]
	PIN1	Phosphorylation-dependent prolyne isomerase	Cellular differentiation; Cellular proliferation; Immune response; Mitosis	[47,48]
	CDC27	Component of anaphase promoting complex	Mitosis; Ubiquitination	[49]
	ANAPC2	Anaphase promoting complex	Cell cycle control; Ubiquitination	[50]
Cell cycle regulation and differentiation	TYMS	Thymidylate syntase	DNA replication and repair; Mitosis	[51]
	FGFR3	Fibroblast growth factor receptor 3	Mitosis; MSCs differentiation	[52,53]
	NCAPD2	Subunit of condensin I	Mitosis; Proliferation	[54,55]
	MITF	Transcription factor	Cell cycle regulation; Gene expression; Differentiation	[56-58]
Oxidative stress response	RPS3	Ribosomal protein (40s subunit)	DNA damage repair; Kinase activity on NFkB complex	[59]
	NFE2L2	Human basic leucine zipper transcription factor	Oxidative stress response	[60]
	HSP1	Heat-shock protein 1	Protein folding	[61-63]
NFkB signalling	IKBKG	Regulatory subunit of IKK complex	NFkB activation	[64]
	MYL5	Myosin light chain	NFkB activation	[65,66]
Apoptosis	SLC6A4	Membrane serotonin transporter	Apoptosis	[67]
Histonic modification	HAT1	Histone acetyltransferase B	Histone acetylation; Aging; Cellular differentiation	[68]
	HIST1H3C	Member of Histone H3 family	Transcriptional regulation; Cellular differentiation	[69]

### TaqMan Real Time quantitative PCR and western blot: validation of the microarray data

**Figure 5:**
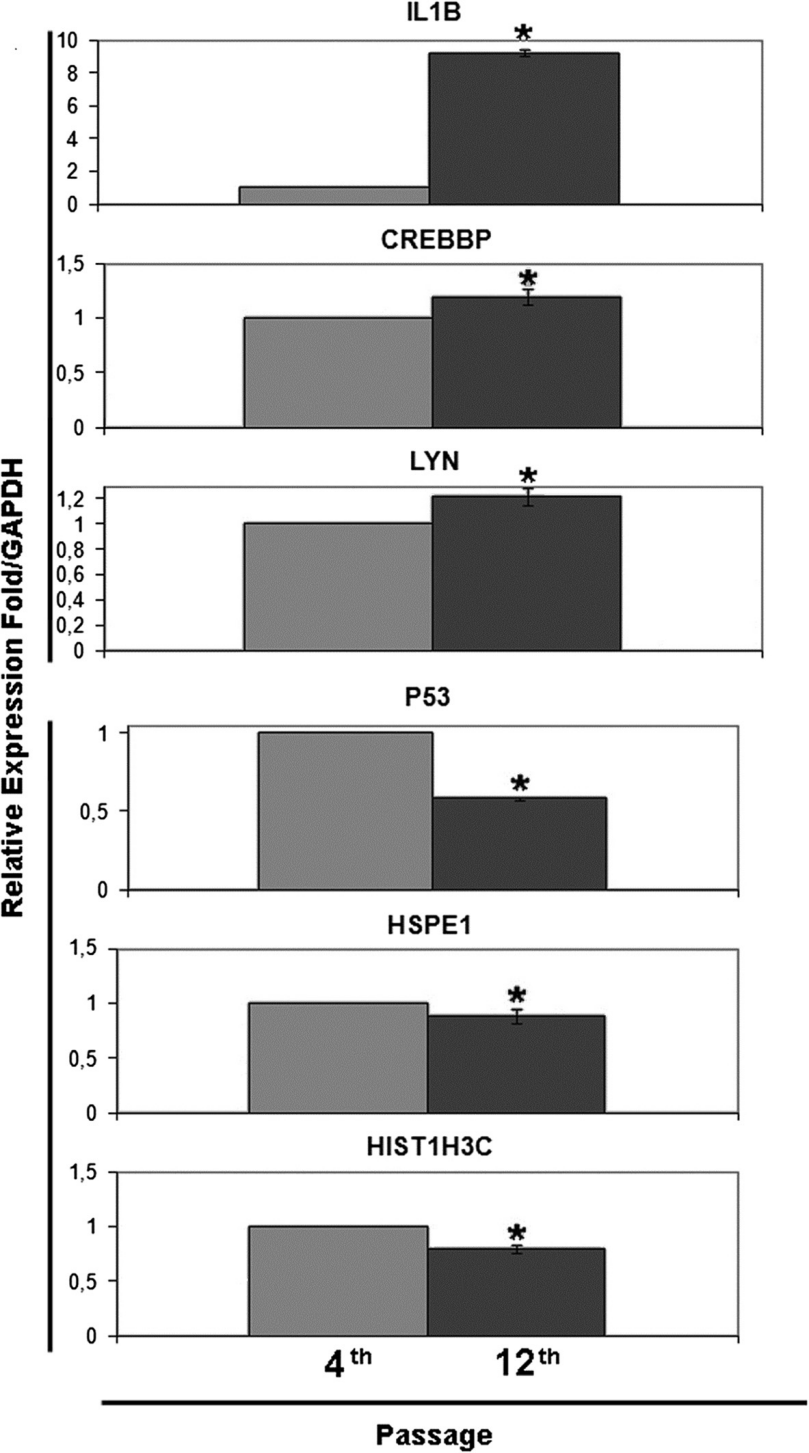
**Validation microarray gene expression data by qRT-PCR.** Analysis of the selected genes IL1B, CREBBP, LYN, P53, HSPE1, HIST1H3C was carried on the RNA obtained by 4^th^ and 12^th^ passage of WJ-MSCs. Data are the means ± SD of three independent experiments in triplicate (*P < 0.01, Student’s t-test).

qRT-PCR analysis, performed in order to validate microarray data by investigating three down-regulated (p53, HSPE1 and HIST1H3C) and three up-regulated genes (IL1B, CREBBP and LYN) present in the first top up and down gene networks, respectively, (Figure 5) and western blot analysis of p53, HIST1H3C and IL1B protein (Additional file 5: Figure S3; Additional file 6: Western Blot Method), confirmed the results obtained by the microarray analysis.

## Discussion

It is well known that during cell culture different kind of stem cells can undergo functional modifications related to the number of culture passages [70,71]. The identification of these modifications is of crucial relevance in order to better understand the biology of stem cells and the differences in their ability to proliferate and differentiate, along with cell expansion. The study of the whole stem cell transcriptome, carried out by microarray technology, allows to identify the global changes occurring in the expression profiles of these cells, providing useful information about their functional changes along with culture progression. In the present study, we carried out an analysis of gene expression profiles of WJ-MSCs at 4^th^ and 12^th^ passages, in order to evidence the modifications in the trascriptome induced by the culture time prolongation. IPA gene analysis revealed that the top up-regulated network was characterized by the presence of 2 independent functional nodes (IL1, AKT). IL1 node is connected with IL1B, IL1R1, S100113 and TWIST2 genes, related to inflammation. The up regulation of these genes in older cells could reflect the presence of an inflammation cellular response acting as a defense mechanism of damaged cells for preventing cell death. AKT node, connected with RAC2 and PTPRF genes, is involved in protein phosphorylation, regulating cellular growth and differentiation, and their over expression appears to be mainly related to cell differentiation and maturation [25]. Other up-regulated genes in this network, although not related to any evident node, are anyway worth of interest due to their specific functions. Among these, four genes (CREBBP, PARP1, HNRNPAB, NONO) are well known to be involved in the regulation of transcription and play an important role in various cellular processes such as differentiation and proliferation [30,32]. In particular, CREBBP gene is critical in embryonic neural development [29] and HNRNPAB gene, interacting with Oct4, is involved in embryonic stem cells differentiation [31]. On the other hand, cytoskeleton organization is the main function played by the other two genes present in the network, complex Arp2/3 and Nischarin (ACTR3, NISCH). In the top up-regulated network are also presents 3 genes (EPHA4, MT2A, CDH2) representing neural marker related to MSC differentiation. Interestingly, the contemporary activation of RAC2 and CDH2 has been reported as related to MSC differentiation [37]. The last three genes with interesting function detected in this network are ERRFI1, RNF7 and LYN. The up-regulation of these genes is associated with cell growth and proliferation [40] and ERRFI1 activity is induced during cell stress [38,72]. RNF7 has an antiapoptotic activity mediated by the activation of the transcription factor JUN [39]. Among the down-regulated gene network, the most interesting node is centered around the TP53 gene, encoding for the major suppressor protein that can recognize DNA damage and subsequently arrest the cell cycle and trigger the repair process. It has been reported that TP53 functional activity is reduced during the aging process [41,42], probably due to a reduced transcriptional activity [73]. In the present study, the down-regulation of the TP53 in older cells was associated to a contemporary down regulation of genes encoding for both transcriptional factors and proteins involved in post-translational modifications, such as UBE2D3, MAP2K3, ZHX1, XPO1, PIN1, CDC27, ANAPC2 [74]. In the same network are also present other genes related to specific cell functions not linked to any evident node gene. Some of these genes, listed in results section, are of particular interest for further discussion. The down regulation of genes related to oxidative stress response (RPS3, NFE2L2, HSP1 and HAT1), has been reported to indicate the cell inability to react to oxidative and DNA damage [59,60,75]. The reduced capacity in protein folding, related to HSP1 down-expression, causes incapacity to react to stress and accelerates aging process [61-63]. The down-expression of IKBKG and MYL5, involved in NF-κB signaling [65], can be related to abnormal growth of cells while MITF down-regulation is associated with loss of pluripotency [56]. In the down-expressed dataset are present two genes (HIST1H3C and HAT1) involved in histone modifications. The down expression of HIST1H3C (encoding a member of the histone H3 family) is related to loss of pluripotency and represents a marker of differentiation in stem cells [76]. The highest expression of HAT1 (encoding a protein involved in rapid acetylation of newly synthesized cytoplasmic histones) occurs during embryogenesis and its down expression is related to aging and differentiation processes [68]. Taken together, all data obtained in this study indicate that WJ-MSCs appear to undergo a process of aging rather than senescence during the in vitro expansion from the 4^th^ to the 12^th^ culture passage. In fact, cellular aging can be defined as a progressive decline in the physiological properties of tissues, characterized by a decreased replication capacity and an increase of cell-cycle-arrested cells, while senescence is the state in which cells have irreversibly lost their proliferation ability [41]. This is mainly demonstrated by the observed low frequency of cells staining positive for β-galactosidase found in all different passages, indicating that even at 12^th^ passage cells cannot be considered as senescent. In addition, further evidences are provided by the presence of intact G1 and G2 cell-cycle checkpoints and by the presence of long telomeric end at all examined passages, which confirm that WJ-MSCs preserve their capability to undergo a high number of cellular divisions up to the 12^th^ passage. Cellular aging can be considered as an evolutionary conserved defence representing an alternative to cell death in the presence of chronic low stress conditions increasing resistance to apoptosis and thus allowing the survival of post-mitotic cells damaged in their central functions. These figures reflects the cell biological condition and confirms previously reported data about the presence of an aging phenotype of these cells as evidenced by proteomic analysis [15]. Salminen et al. [41], have recently described that the molecular basis of increased resistance to apoptosis in aging cells involves several mechanisms such as alterations in p53 and NF-κB networks and pathways, protein folding, and increased presence of pro-inflammatory mediators. Our results match with these mechanisms, demonstrating a resistance to apoptosis related to the above described down-expression of p53 and the over-expression of TWIST2, RNF7 and ILB1 genes, present in the first top network, as well as of SOD2, RPS27L and STAMBP genes, included in the up-expressed gene cluster. TWIST2 and ILB1 are able to induce the resistance to apoptosis mediated by NF-κB [77], which activates anti-apoptotic survival genes such as SOD2, the cytokine STAMBP, and the caspase activator RPS27L, all positive regulator of anti-apoptotic signaling acting on different pathways. Feng et al. [74], convincingly demonstrated that the functional activity of p53 declines in several murine tissues during aging. Moreover, a reduced capacity in protein folding is related to apoptosis and cell death. Aging process and incapacity to react to stress are also related to down expression of the NFE2L2-NFE2-NFE2L gene complex [60]. The resistance to apoptosis is also enhanced by persistent type of stress, e.g. oxidative stress [41] and in this view, our data show the over-expression of IL1B, LTBP1 AND RAC2 genes, related to the production of oxidative species. The aging process denotes a reduced capacity to maintain intact cytoskeleton structures, affecting cellular processes such as motility, interaction with neighbouring cells and mitosis [34], and in the present study, we demonstrated the over-expression in older cells of NISCH gene. ACTR3 instead is demonstrated to be up-regulated during stem cell differentiation [33], in particular the neural differentiation. Moreover, as confirmed by data from cell cultures, the cells at the 12^th^ passage show decreased proliferation likely related to the above described down-expression of genes such as MAP2K3, PIN1, CDC27, ANAPC2, TYMS, FGFR3, NCAPD2, MYL5. The same cells also show altered modulation of HIST1H3C, PTPRF, EPHA4, MT2A, CDK5R2, CDH2, genes related to neuronal differentiation indicating a loss in WJ-MSCs plasticity.

## Conclusion

In conclusion, data provided by cell culture experiments and results obtained by profiling studies, all together demonstrate the impairment WJ-MSCs expansion abilities and their resistance to apoptosis, two hallmarks of cell aging. On the other hand, all these data show the need to develop novel culture protocols able to preserve stem cell plasticity. Moreover, the genes identified as impaired in the present study, could be useful biomarkers to evaluate cell culture quality when comparing different in vitro expansion methods.

## Availability of supporting data

Raw data of the performed microarray experiments have been recorded in the GEO public database (accession number: GSE34929).

## Competing interests

No competing financial interests exist.

## Authors’ contributions

VG participated in the design of the study and performed the assembly of data, data analysis and interpretation and drafted the manuscript. MDA participated in assembly of data, microarray experiments, manuscript writing. PL performed cell isolation and culture and telomere length assay. LP performed cell isolation and culture and cell differentiation experiments. SS carried out microarray experiments and participated in assembly of data. GP participated to data analysis and interpretation. MG participated to data analysis and interpretation. MM conceived of the study and participated to samples collection and data interpretation. SM participated in study design and coordination and revising the manuscript critically for important intellectual content. LS participated in study design and coordination, data interpretation and helped to final approval of the manuscript. All authors read and approved the final manuscript.

## Authors’ information

Sebastiano Miscia and Liborio Stuppia senior investigators contributed equally to this work.

## Supplementary Material

Additional file 1: Figure S1 Flow cytometric analyses of surface markers at 4^th^ and 12^th^ passage of WJ-MSC. Flow cytometric analysis of WJ-MSCs surface antigen expression profile: CD13, CD14, CD29, CD34, CD44, CD45, CD73, CD90, CD105, CD117 CD133, CD146, CD166, HLA-ABC and HLA-DR Filled histograms represent cells stained with the expression markers; empty histograms show the respective IgG isotype controls. Data are representative of five separate biological samples. Click here for file

Additional file 2: Figure S2 Flow cytometric analysis of WJ-MSC doubling time and cell cycle. Representative flow cytometric analysis of WJ-MSC doubling time, evaluated by the BrdU incorporation assay (A) and WJ-MSC cell cycle profile obtained by the PI staining only (B). Data are representative of five separate biological samples. Click here for file

Additional file 3: Table S1 List of transcripts resulting up-expressed in the cluster 1. Click here for file

Additional file 4: Table S2 List of transcripts resulting down-expressed in the cluster 2. Click here for file

Additional file 5: Figure S3 Western blot analysis. The intensity of immune-reactivity bands (10 μg of protein) of HIST1H3C, P53 and IL1β was measured by densitometry analysis, normalized respect to the corresponding β-Actin bands and expressed as arbitrary units (A.U.) (bottom histograms). Data are expressed as the averages ± SD of three independent experiments. Click here for file

Additional file 6 Western Blot Method. Click here for file
