# Supplementary figures and images for: Gene expression modifications in Wharton’s Jelly mesenchymal stem cells promoted by prolonged in vitro culturing
Source: BMC Genomics. 2013 Sep 21;14:635. doi: 10.1186/1471-2164-14-635 (PMC3849041; doi:10.1186/1471-2164-14-635)

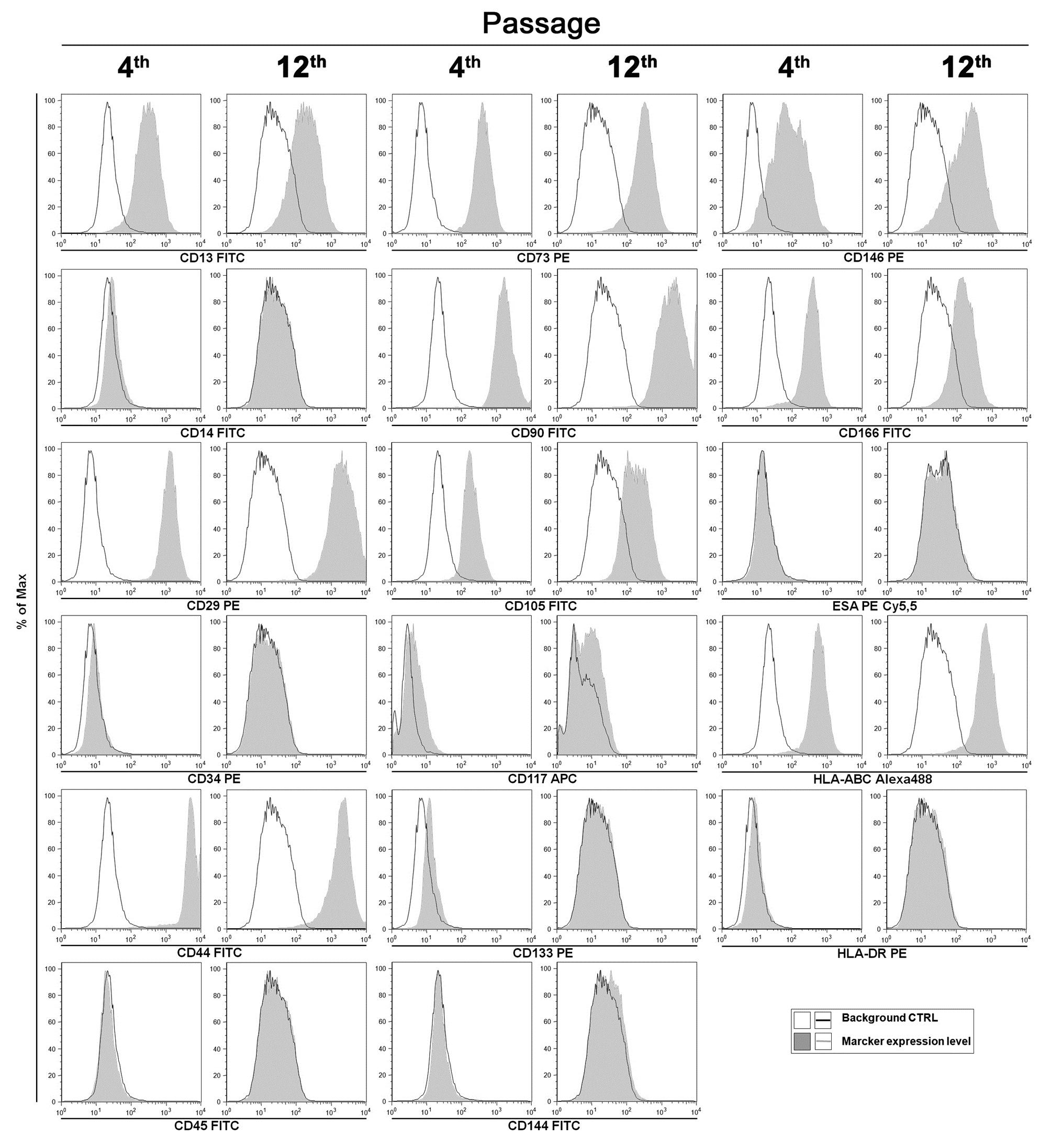

Supplement: Additional file 1: Figure S1 — Flow cytometric analyses of surface markers at 4th and 12th passage of WJ-MSC. Flow cytometric analysis of WJ-MSCs surface antigen expression profile: CD13, CD14, CD29, CD34, CD44, CD45, CD73, CD90, CD105, CD117 CD133, CD146, CD166, HLA-ABC and HLA-DR Filled histograms represent cells stained with the expression markers; empty histograms show the respective IgG isotype controls. Data are representative of five separate biological samples. [file 1471-2164-14-635-S1.jpeg]

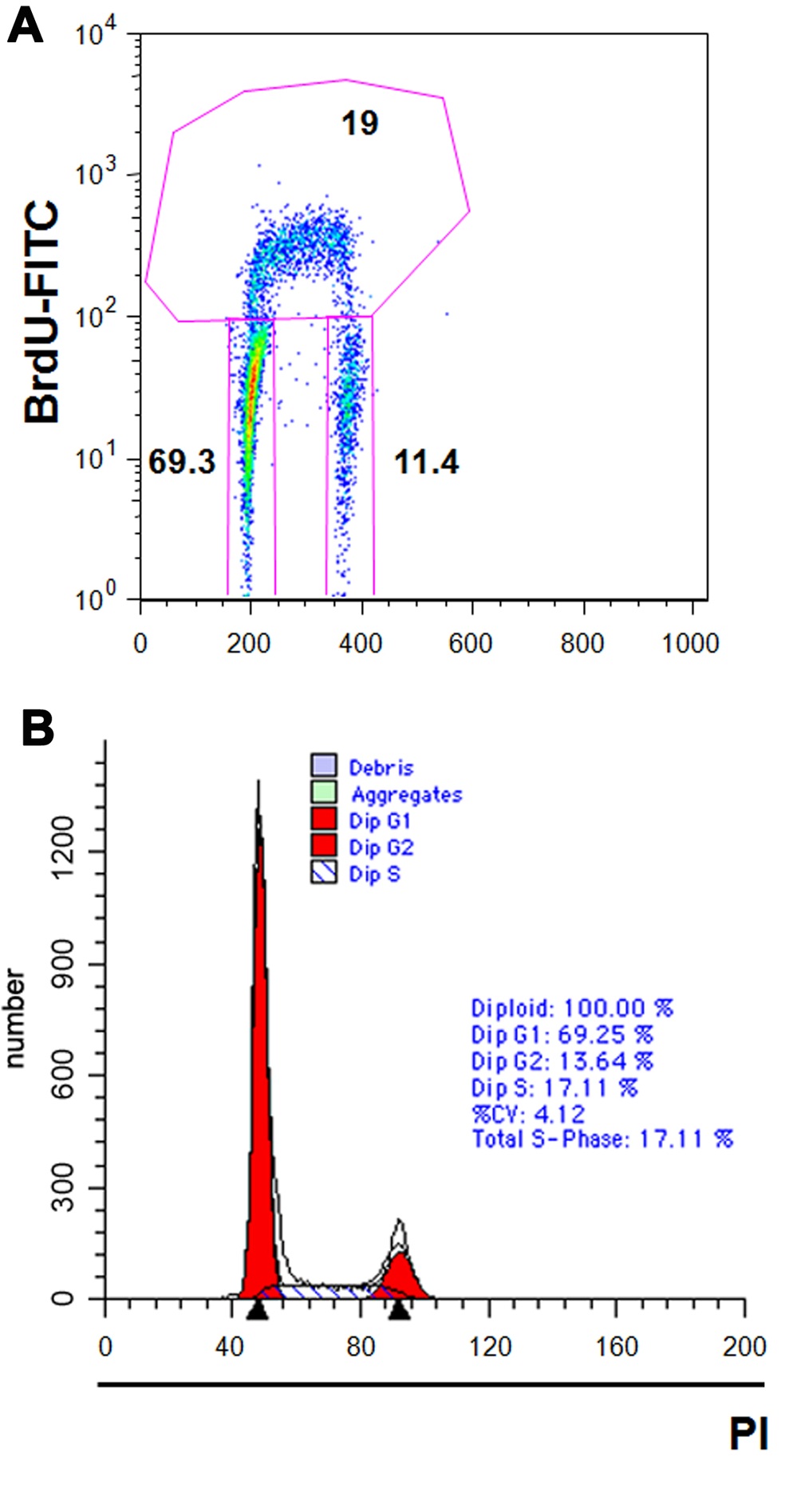

Supplement: Additional file 2: Figure S2 — Flow cytometric analysis of WJ-MSC doubling time and cell cycle. Representative flow cytometric analysis of WJ-MSC doubling time, evaluated by the BrdU incorporation assay (A) and WJ-MSC cell cycle profile obtained by the PI staining only (B). Data are representative of five separate biological samples. [file 1471-2164-14-635-S2.jpeg]

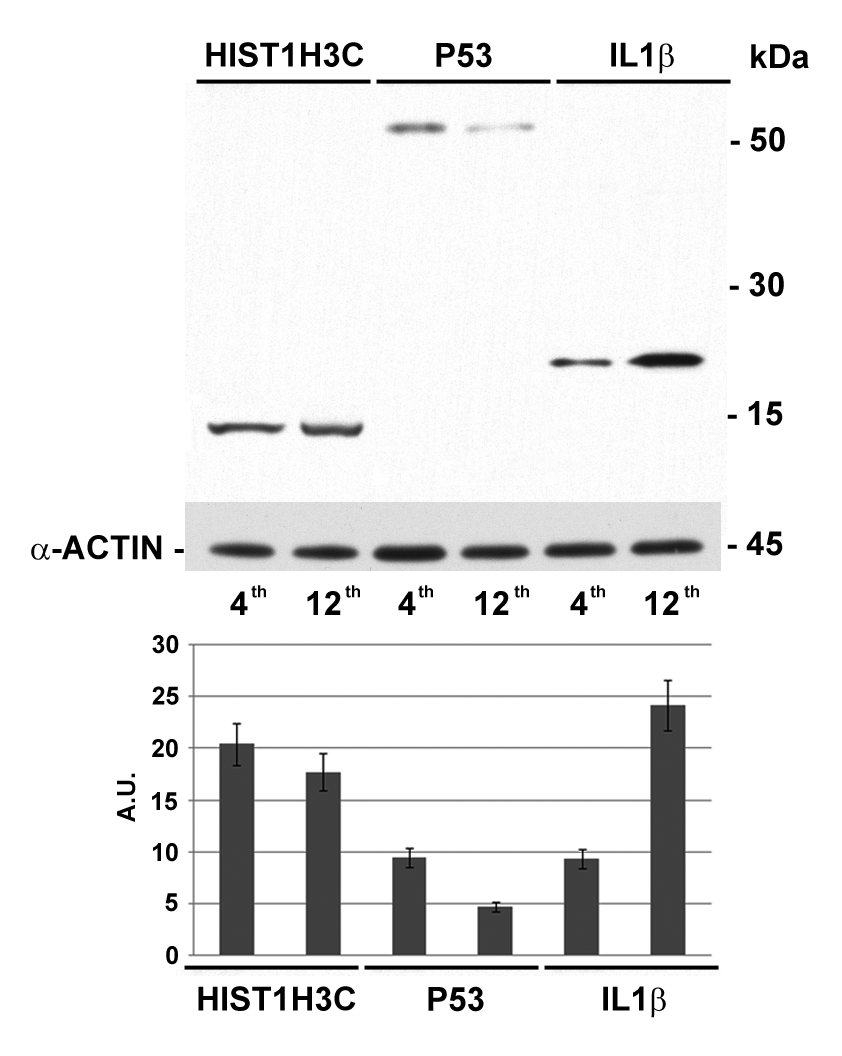

Supplement: Additional file 5: Figure S3 — Western blot analysis. The intensity of immune-reactivity bands (10 μg of protein) of HIST1H3C, P53 and IL1β was measured by densitometry analysis, normalized respect to the corresponding β-Actin bands and expressed as arbitrary units (A.U.) (bottom histograms). Data are expressed as the averages ± SD of three independent experiments. [file 1471-2164-14-635-S5.tiff]
