# Supplementary material for: Gene expression modifications in Wharton’s Jelly mesenchymal stem cells promoted by prolonged in vitro culturing
Source: BMC Genomics. 2013 Sep 21;14:635. doi: 10.1186/1471-2164-14-635 (PMC3849041; doi:10.1186/1471-2164-14-635)
Supplement: Additional file 3: Table S1 — List of transcripts resulting up-expressed in the cluster 1. [file 1471-2164-14-635-S3.docx]

| **Supplemental Table 1:** List of transcripts resulting up-expressed in the cluster 1. | | | | |
| --- | --- | --- | --- | --- |
| ID | Symbol | Entrez Gene Name | Location | Type |
| NM_005721.3 | ACTR3 | ARP3 actin-related protein 3 homolog (yeast) | Plasma Membrane | other |
| NM_001110.1 | ADAM10 | ADAM metallopeptidase domain 10 | Plasma Membrane | peptidase |
| NM_003815.3 | ADAM15 | ADAM metallopeptidase domain 15 | Plasma Membrane | peptidase |
| BM696171 | ALKBH7 | alkB, alkylation repair homolog 7 (E. coli) | Cytoplasm | other |
| XM_380018.2 | ANKIB1 | ankyrin repeat and IBR domain containing 1 | Nucleus | transcription regulator |
| BX649010 | APPL2 | adaptor protein, phosphotyrosine interaction, PH domain and leucine zipper containing 2 | Cytoplasm | other |
| NM_014882.2 | ARHGAP25 | Rho GTPase activating protein 25 | Cytoplasm | other |
| NM_019007.2 | ARMCX6 | armadillo repeat containing, X-linked 6 | unknown | other |
| NM_001696.2 | ATP6V1E1 | ATPase, H+ transporting, lysosomal 31kDa, V1 subunit E1 | Cytoplasm | transporter |
| NM_004776.2 | B4GALT5 | UDP-Gal:betaGlcNAc beta 1,4- galactosyltransferase, polypeptide 5 | Cytoplasm | enzyme |
| NM_006698.2 | BLCAP | bladder cancer associated protein | unknown | other |
| NM_000060.1 | BTD | biotinidase | Extracellular Space | enzyme |
| NM_004894.1 | C14orf2 | chromosome 14 open reading frame 2 | unknown | other |
| NM_020154.1 | C15orf24 | chromosome 15 open reading frame 24 | Extracellular Space | other |
| NM_015449.1 | C1orf43 | chromosome 1 open reading frame 43 | unknown | other |
| NM_001212.3 | C1QBP | complement component 1, q subcomponent binding protein | Cytoplasm | other |
| NM_138423.2 | CASC4 | cancer susceptibility candidate 4 | unknown | other |
| NM_004357.3 | CD151 | CD151 molecule (Raph blood group) | Plasma Membrane | other |
| NM_003607.2 | CDC42BPA | CDC42 binding protein kinase alpha (DMPK-like) | Cytoplasm | kinase |
| NM_001792.2 | CDH2 | cadherin 2, type 1, N-cadherin (neuronal) | Plasma Membrane | other |
| NM_004344.1 | CETN2 | centrin, EF-hand protein, 2 | Nucleus | enzyme |
| NM_001280.1 | CIRBP | cold inducible RNA binding protein | Nucleus | translation regulator |
| NM_020188.2 | CMC2 (includes EG:368857) | COX assembly mitochondrial protein 2 homolog (S. cerevisiae) | Cytoplasm | other |
| NM_001861.2 | COX4I1 | cytochrome c oxidase subunit IV isoform 1 | Cytoplasm | enzyme |
| NM_004074.2 | COX8A | cytochrome c oxidase subunit VIIIA (ubiquitous) | Cytoplasm | enzyme |
| NM_016352.1 | CPA4 | carboxypeptidase A4 | Extracellular Space | peptidase |
| NM_004380.1 | CREBBP | CREB binding protein | Nucleus | transcription regulator |
| NM_018947.4 | CYCS | cytochrome c, somatic | Cytoplasm | enzyme |
| NM_001417.2 | EIF4B | eukaryotic translation initiation factor 4B | Cytoplasm | translation regulator |
| NM_021814.3 | ELOVL5 | ELOVL fatty acid elongase 5 | Cytoplasm | enzyme |
| NM_001247.1 | ENTPD6 | ectonucleoside triphosphate diphosphohydrolase 6 (putative) | Cytoplasm | enzyme |
| NM_015630.2 | EPC2 | enhancer of polycomb homolog 2 (Drosophila) | unknown | other |
| NM_004438.3 | EPHA4 | EPH receptor A4 | Plasma Membrane | kinase |
| NM_018948.2 | ERRFI1 | ERBB receptor feedback inhibitor 1 | Cytoplasm | other |
| NM_004730.1 | ETF1 | eukaryotic translation termination factor 1 | Cytoplasm | translation regulator |
| NM_016255.1 | FAM8A1 | family with sequence similarity 8, member A1 | unknown | other |
| NM_006832.1 | FERMT2 | fermitin family member 2 | Cytoplasm | other |
| NM_001457.1 | FLNB | filamin B, beta | Cytoplasm | other |
| BM678420 | FOXP2 | forkhead box P2 | Nucleus | transcription regulator |
| NM_007085.3 | FSTL1 | follistatin-like 1 | Extracellular Space | other |
| NM_021167.2 | GATAD1 | GATA zinc finger domain containing 1 | unknown | other |
| NM_000165.2 | GJA1 | gap junction protein, alpha 1, 43kDa | Plasma Membrane | transporter |
| NM_014905.2 | GLS | glutaminase | Cytoplasm | enzyme |
| NM_015530.3 | GORASP2 | golgi reassembly stacking protein 2, 55kDa | Cytoplasm | other |
| NM_003979.2 | GPRC5A | G protein-coupled receptor, family C, group 5, member A | Plasma Membrane | G-protein coupled receptor |
| NM_012257.3 | HBP1 | HMG-box transcription factor 1 | Nucleus | transcription regulator |
| NM_005487.3 | HMGXB4 | HMG box domain containing 4 | Nucleus | other |
| NM_031266.2 | HNRNPAB | heterogeneous nuclear ribonucleoprotein A/B | Nucleus | enzyme |
| NM_022081.4 | HPS4 (includes EG:192232) | Hermansky-Pudlak syndrome 4 | Cytoplasm | other |
| NM_000576.2 | IL1B | interleukin 1, beta | Extracellular Space | cytokine |
| NM_000877.2 | IL1R1 | interleukin 1 receptor, type I | Plasma Membrane | transmembrane receptor |
| NM_000884.1 | IMPDH2 | IMP (inosine 5'-monophosphate) dehydrogenase 2 | Cytoplasm | enzyme |
| NM_002194.2 | INPP1 | inositol polyphosphate-1-phosphatase | Cytoplasm | phosphatase |
| NM_002265.4 | KPNB1 | karyopherin (importin) beta 1 | Nucleus | transporter |
| NM_014017.1 | LAMTOR2 | late endosomal/lysosomal adaptor, MAPK and MTOR activator 2 | Cytoplasm | other |
| NM_002300.3 | LDHB | lactate dehydrogenase B | Cytoplasm | enzyme |
| NM_000527.2 | LDLR | low density lipoprotein receptor | Plasma Membrane | transporter |
| NM_014262.2 | LEPREL2 | leprecan-like 2 | Nucleus | enzyme |
| NM_178175.2 | LHFPL1 | lipoma HMGIC fusion partner-like 1 | unknown | other |
| NM_152271.2 | LONRF1 | LON peptidase N-terminal domain and ring finger 1 | unknown | other |
| NM_015578.1 | LSM14A | LSM14A, SCD6 homolog A (S. cerevisiae) | Cytoplasm | other |
| NM_000627.2 | LTBP1 | latent transforming growth factor beta binding protein 1 | Extracellular Space | other |
| NM_002350.1 | LYN | v-yes-1 Yamaguchi sarcoma viral related oncogene homolog | Cytoplasm | kinase |
| AK074092 | MAGED1 | melanoma antigen family D, 1 | Plasma Membrane | transcription regulator |
| BX647087 | MGAT5 | mannosyl (alpha-1,6-)-glycoprotein beta-1,6-N-acetyl-glucosaminyltransferase | Cytoplasm | enzyme |
| NM_173468.2 | MOB1B | MOB kinase activator 1B | Cytoplasm | other |
| NM_016491.2 | MRPL37 (includes EG:51253) | mitochondrial ribosomal protein L37 | Cytoplasm | enzyme |
| NM_014046.2 | MRPS18B | mitochondrial ribosomal protein S18B | Cytoplasm | other |
| NM_032014.1 | MRPS24 | mitochondrial ribosomal protein S24 | Cytoplasm | other |
| NM_033281.4 | MRPS36 | mitochondrial ribosomal protein S36 | Cytoplasm | other |
| NM_005953.2 | MT2A | metallothionein 2A | Cytoplasm | other |
| NM_145808.1 | MTPN | myotrophin | Nucleus | transcription regulator |
| NM_006471.2 | MYL12A | myosin, light chain 12A, regulatory, non-sarcomeric | unknown | other |
| NM_018838.3 | NDUFA12 | NADH dehydrogenase (ubiquinone) 1 alpha subcomplex, 12 | Cytoplasm | enzyme |
| NM_004552.1 | NDUFS5 | NADH dehydrogenase (ubiquinone) Fe-S protein 5, 15kDa (NADH-coenzyme Q reductase) | Cytoplasm | enzyme |
| NM_004553.2 | NDUFS6 | NADH dehydrogenase (ubiquinone) Fe-S protein 6, 13kDa (NADH-coenzyme Q reductase) | Cytoplasm | enzyme |
| NM_007184.1 | NISCH | nischarin | Plasma Membrane | transmembrane receptor |
| NM_007363.3 | NONO | non-POU domain containing, octamer-binding | Nucleus | other |
| NM_003580.2 | NSMAF | neutral sphingomyelinase (N-SMase) activation associated factor | Cytoplasm | other |
| NM_017602.2 | OTUD5 | OTU domain containing 5 | Cytoplasm | enzyme |
| BF511864 | PAEP | progestagen-associated endometrial protein | Extracellular Space | other |
| NM_000430.2 | PAFAH1B1 | platelet-activating factor acetylhydrolase 1b, regulatory subunit 1 (45kDa) | Cytoplasm | enzyme |
| NM_001618.2 | PARP1 | poly (ADP-ribose) polymerase 1 | Nucleus | enzyme |
| NM_001122.2 | PLIN2 | perilipin 2 | Plasma Membrane | other |
| NM_022572.2 | PNKD | paroxysmal nonkinesigenic dyskinesia | Nucleus | other |
| NM_052926.1 | PNMA5 | paraneoplastic Ma antigen family member 5 | unknown | other |
| NM_015932.2 | POMP | proteasome maturation protein | Nucleus | other |
| NM_006245.2 | PPP2R5D | protein phosphatase 2, regulatory subunit B', delta | Nucleus | phosphatase |
| NM_145040.2 | PRKCDBP | protein kinase C, delta binding protein | Cytoplasm | other |
| NM_013270.2 | PRSS50 | protease, serine, 50 | Cytoplasm | peptidase |
| NM_002808.3 | PSMD2 | proteasome (prosome, macropain) 26S subunit, non-ATPase, 2 | Cytoplasm | other |
| NM_002840.2 | PTPRF | protein tyrosine phosphatase, receptor type, F | Plasma Membrane | phosphatase |
| NM_005704.2 | PTPRU | protein tyrosine phosphatase, receptor type, U | Plasma Membrane | phosphatase |
| NM_002852.2 | PTX3 | pentraxin 3, long | Extracellular Space | other |
| NM_002872.3 | RAC2 | ras-related C3 botulinum toxin substrate 2 (rho family, small GTP binding protein Rac2) | Cytoplasm | enzyme |
| AK093863 | RBMS3 | RNA binding motif, single stranded interacting protein 3 | unknown | other |
| NM_004040.2 | RHOB | ras homolog family member B | Cytoplasm | enzyme |
| NM_014245.2 | RNF7 | ring finger protein 7 | Nucleus | enzyme |
| NM_000975.2 | RPL11 | ribosomal protein L11 | Cytoplasm | other |
| NM_001023.2 | RPS20 | ribosomal protein S20 | Cytoplasm | other |
| NM_015920.2 | RPS27L | ribosomal protein S27-like | Nucleus | other |
| NM_012424.2 | RPS6KC1 | ribosomal protein S6 kinase, 52kDa, polypeptide 1 | Cytoplasm | kinase |
| NM_014328.2 | RUSC1 | RUN and SH3 domain containing 1 | Cytoplasm | other |
| XM_371380.2 | S100A13 | S100 calcium binding protein A13 | Cytoplasm | other |
| NM_153334.3 | SCARF2 | scavenger receptor class F, member 2 | Plasma Membrane | transmembrane receptor |
| NM_014300.2 | SEC11A | SEC11 homolog A (S. cerevisiae) | Cytoplasm | peptidase |
| NM_006808.2 | SEC61B | Sec61 beta subunit | Cytoplasm | transporter |
| NM_021627.2 | SENP2 | SUMO1/sentrin/SMT3 specific peptidase 2 | Nucleus | peptidase |
| NM_031459.3 | SESN2 | sestrin 2 | Cytoplasm | other |
| NM_152679.2 | SLC10A4 | solute carrier family 10 (sodium/bile acid cotransporter family), member 4 | Plasma Membrane | transporter |
| NM_003093.1 | SNRPC | small nuclear ribonucleoprotein polypeptide C | Nucleus | other |
| NM_000636.1 | SOD2 | superoxide dismutase 2, mitochondrial | Cytoplasm | enzyme |
| NM_006463.3 | STAMBP | STAM binding protein | Nucleus | enzyme |
| NM_182760.2 | SUMF1 | sulfatase modifying factor 1 | Cytoplasm | other |
| NM_014604.1 | TAX1BP3 | Tax1 (human T-cell leukemia virus type I) binding protein 3 | Nucleus | transcription regulator |
| NM_003234.1 | TFRC | transferrin receptor (p90, CD71) | Plasma Membrane | transporter |
| NM_006335.1 | TIMM17A | translocase of inner mitochondrial membrane 17 homolog A (yeast) | Cytoplasm | transporter |
| NM_000362.3 | TIMP3 | TIMP metallopeptidase inhibitor 3 | Extracellular Space | other |
| NM_031298.1 | TMEM93 | transmembrane protein 93 | unknown | other |
| NM_015939.3 | TRMT6 | tRNA methyltransferase 6 homolog (S. cerevisiae) | unknown | other |
| NM_021733.1 | TSKS | testis-specific serine kinase substrate | Cytoplasm | kinase |
| NM_057179.1 | TWIST2 | twist homolog 2 (Drosophila) | Nucleus | transcription regulator |
| NM_181291.1 | WDR20 | WD repeat domain 20 | unknown | other |
| NM_025234.1 | WDR61 | WD repeat domain 61 | unknown | other |
| NM_003405.2 | YWHAH | tyrosine 3-monooxygenase/tryptophan 5-monooxygenase activation protein, eta polypeptide | Cytoplasm | transcription regulator |
| NM_021211.2 | ZBED5 | zinc finger, BED-type containing 5 | Nucleus | other |
| NM_016598.1 | ZDHHC3 | zinc finger, DHHC-type containing 3 | Cytoplasm | peptidase |
| NM_053023.2 | ZFP91 | zinc finger protein 91 homolog (mouse) | Nucleus | transcription regulator |
| NM_001002836.1 | ZNF787 | zinc finger protein 787 | unknown | other |
| NM_005455.3 | ZRANB2 | zinc finger, RAN-binding domain containing 2 | Nucleus | transcription regulator |
| NA | unknown | unknown | unknown | unknown |
| XM_378224.2 | unknown | unknown | unknown | unknown |
| NA | unknown | unknown | unknown | unknown |
| NA | unknown | unknown | unknown | unknown |
| NA | unknown | unknown | unknown | unknown |
| NA | unknown | unknown | unknown | unknown |
| NA | unknown | unknown | unknown | unknown |
| NA | unknown | unknown | unknown | unknown |
| NA | unknown | unknown | unknown | unknown |
| XM_379885.2 | unknown | unknown | unknown | unknown |
| NA | unknown | unknown | unknown | unknown |
| NA | unknown | unknown | unknown | unknown |
| NA | unknown | unknown | unknown | unknown |
| XM_209423.4 | unknown | unknown | unknown | unknown |
| NA | unknown | unknown | unknown | unknown |
| XM_376838.2 | unknown | unknown | unknown | unknown |
| NA | unknown | unknown | unknown | unknown |
| XM_376043.2 | unknown | unknown | unknown | unknown |
| BU608039 | unknown | unknown | unknown | unknown |
| XM_496629.1 | unknown | unknown | unknown | unknown |
| NA | unknown | unknown | unknown | unknown |
| BM472931 | unknown | unknown | unknown | unknown |
| BE501535 | unknown | unknown | unknown | unknown |
| NA | unknown | unknown | unknown | unknown |
| BQ018505 | unknown | unknown | unknown | unknown |
| XM_497120.1 | unknown | unknown | unknown | unknown |
| BG201274 | unknown | unknown | unknown | unknown |
