# Supplementary material for: Gene expression modifications in Wharton’s Jelly mesenchymal stem cells promoted by prolonged in vitro culturing
Source: BMC Genomics. 2013 Sep 21;14:635. doi: 10.1186/1471-2164-14-635 (PMC3849041; doi:10.1186/1471-2164-14-635)
Supplement: Additional file 4: Table S2 — List of transcripts resulting down-expressed in the cluster 2. [file 1471-2164-14-635-S4.docx]

| **Supplemental Table 2:** List of transcripts resulting down-expressed in the cluster 2. | | | | |
| --- | --- | --- | --- | --- |
| NM_016006.3 | ABHD5 | abhydrolase domain containing 5 | Cytoplasm | enzyme |
| NM_014945.1 | ABLIM3 | actin binding LIM protein family, member 3 | Cytoplasm | other |
| NM_001104.1 | ACTN3 | actinin, alpha 3 | Cytoplasm | other |
| NM_139054.2 | ADAMTS18 | ADAM metallopeptidase with thrombospondin type 1 motif, 18 | Extracellular Space | peptidase |
| NM_024876.2 | ADCK4 | aarF domain containing kinase 4 | Cytoplasm | kinase |
| NM_014914.2 | AGAP1 | ArfGAP with GTPase domain, ankyrin repeat and PH domain 1 | Cytoplasm | enzyme |
| NM_032878.2 | ALKBH6 | alkB, alkylation repair homolog 6 (E. coli) | unknown | other |
| NM_130847.1 | AMOTL1 | angiomotin like 1 | Plasma Membrane | other |
| NM_013366.3 | ANAPC2 | anaphase promoting complex subunit 2 | Nucleus | enzyme |
| NM_144994.6 | ANKRD23 | ankyrin repeat domain 23 | Nucleus | other |
| XM_114000.4 | ANKRD24 | ankyrin repeat domain 24 | unknown | other |
| NM_005224.1 | ARID3A | AT rich interactive domain 3A (BRIGHT-like) | Nucleus | transcription regulator |
| NM_024742.1 | ARMC5 | armadillo repeat containing 5 | unknown | other |
| NM_177949.1 | ARMCX2 | armadillo repeat containing, X-linked 2 | unknown | other |
| CR627251 | ARRDC1 | arrestin domain containing 1 | unknown | other |
| NM_198186.2 | ASTN2 | astrotactin 2 | unknown | other |
| NM_015459.3 | ATL3 | atlastin GTPase 3 | Cytoplasm | other |
| NM_000333.2 | ATXN7 | ataxin 7 | Nucleus | other |
| NM_020371.1 | AVEN | apoptosis, caspase activation inhibitor | Cytoplasm | other |
| XM_088691.4 | AWAT1 | acyl-CoA wax alcohol acyltransferase 1 | unknown | other |
| NM_030578.2 | B9D2 | B9 protein domain 2 | Cytoplasm | other |
| BU729871 | BOLA1 | bolA homolog 1 (E. coli) | Cytoplasm | other |
| BC067086 | BTN3A2 | butyrophilin, subfamily 3, member A2 | unknown | other |
| NM_021830.3 | C10orf2 | chromosome 10 open reading frame 2 | Cytoplasm | enzyme |
| AK056213 | C12orf60 | chromosome 12 open reading frame 60 | unknown | other |
| NM_207440.1 | C13orf35 | chromosome 13 open reading frame 35 | unknown | other |
| NM_173608.1 | C14orf80 | chromosome 14 open reading frame 80 | unknown | other |
| NM_021944.1 | C14orf93 | chromosome 14 open reading frame 93 | unknown | other |
| BE792494 | C16orf42 | chromosome 16 open reading frame 42 | unknown | other |
| NM_024648.1 | C17orf101 | chromosome 17 open reading frame 101 | unknown | enzyme |
| NM_145278.1 | C1orf150 | chromosome 1 open reading frame 150 | unknown | other |
| NM_152608.2 | C1orf55 | chromosome 1 open reading frame 55 | unknown | other |
| NM_181435.2 | C1QTNF3 | C1q and tumor necrosis factor related protein 3 | Extracellular Space | other |
| NM_198562.1 | C3orf62 | chromosome 3 open reading frame 62 | unknown | other |
| AK096130 | CCDC150 | coiled-coil domain containing 150 | unknown | other |
| NM_024296.2 | CCDC28B | coiled-coil domain containing 28B | unknown | other |
| NM_024768.1 | CCDC48 | coiled-coil domain containing 48 | unknown | other |
| NM_198082.1 | CCDC57 | coiled-coil domain containing 57 | unknown | other |
| NM_014880.3 | CD302 | CD302 molecule | Plasma Membrane | transmembrane receptor |
| NM_001256.2 | CDC27 | cell division cycle 27 homolog (S. cerevisiae) | Nucleus | other |
| NM_007061.3 | CDC42EP1 | CDC42 effector protein (Rho GTPase binding) 1 | Extracellular Space | other |
| NM_004933.2 | CDH15 | cadherin 15, type 1, M-cadherin (myotubule) | Plasma Membrane | other |
| NM_033100.1 | CDHR1 | cadherin-related family member 1 | Plasma Membrane | other |
| NM_003936.3 | CDK5R2 | cyclin-dependent kinase 5, regulatory subunit 2 (p39) | Nucleus | other |
| NM_018451.2 | CENPJ | centromere protein J | Nucleus | transcription regulator |
| BM695642 | CES5AP1 | carboxylesterase 5A pseudogene 1 | unknown | other |
| NM_014453.2 | CHMP2A | charged multivesicular body protein 2A | Cytoplasm | other |
| NM_139320.1 | CHRFAM7A | CHRNA7 (cholinergic receptor, nicotinic, alpha 7, exons 5-10) and FAM7A (family with sequence similarity 7A, exons A-E) fusion | unknown | other |
| NM_199328.1 | CLDN8 | claudin 8 | Plasma Membrane | other |
| NM_014900.2 | COBLL1 | COBL-like 1 | unknown | other |
| NM_001855.2 | COL15A1 | collagen, type XV, alpha 1 | Extracellular Space | other |
| NM_003653.2 | COPS3 | COP9 constitutive photomorphogenic homolog subunit 3 (Arabidopsis) | Cytoplasm | other |
| AK096309 | COQ10B | coenzyme Q10 homolog B (S. cerevisiae) | Cytoplasm | other |
| AK023076 | COQ2 | coenzyme Q2 homolog, prenyltransferase (yeast) | Cytoplasm | enzyme |
| NM_014325.2 | CORO1C | coronin, actin binding protein, 1C | Cytoplasm | other |
| NM_022663.1 | CTAGE1 | cutaneous T-cell lymphoma-associated antigen 1 | unknown | other |
| NM_001906.1 | CTRB1 | chymotrypsinogen B1 | Extracellular Space | peptidase |
| NM_001907.1 | CTRL | chymotrypsin-like | Extracellular Space | peptidase |
| NM_005409.3 | CXCL11 | chemokine (C-X-C motif) ligand 11 | Extracellular Space | cytokine |
| XM_098980.6 | CXorf30 | chromosome X open reading frame 30 | unknown | other |
| NM_000500.4 | CYP21A2 | cytochrome P450, family 21, subfamily A, polypeptide 2 | Cytoplasm | enzyme |
| NM_024898.1 | DENND1C | DENN/MADD domain containing 1C | unknown | other |
| NM_032656.2 | DHX37 | DEAH (Asp-Glu-Ala-His) box polypeptide 37 | unknown | enzyme |
| NM_024612.3 | DHX40 | DEAH (Asp-Glu-Ala-His) box polypeptide 40 | unknown | enzyme |
| NM_001003399.1 | DKFZp451A211 | uncharacterized LOC400169 | unknown | other |
| NM_152721.2 | DOK6 | docking protein 6 | unknown | other |
| NM_001382.2 | DPAGT1 | dolichyl-phosphate (UDP-N-acetylglucosamine) N-acetylglucosaminephosphotransferase 1 (GlcNAc-1-P transferase) | Cytoplasm | enzyme |
| NM_000110.2 | DPYD | dihydropyrimidine dehydrogenase | Cytoplasm | enzyme |
| NM_020390.5 | EIF5A2 | eukaryotic translation initiation factor 5A2 | Cytoplasm | translation regulator |
| NM_024090.1 | ELOVL6 | ELOVL fatty acid elongase 6 | Cytoplasm | enzyme |
| NM_001978.1 | EPB49 | erythrocyte membrane protein band 4.9 (dematin) | Plasma Membrane | other |
| NM_004449.3 | ERG | v-ets erythroblastosis virus E26 oncogene homolog (avian) | Nucleus | transcription regulator |
| NM_016570.2 | ERGIC2 | ERGIC and golgi 2 | Cytoplasm | other |
| AK024276 | EXOSC6 | exosome component 6 | Nucleus | other |
| NM_203305.1 | FAM102A | family with sequence similarity 102, member A | unknown | other |
| NM_138371.1 | FAM113B | family with sequence similarity 113, member B | unknown | other |
| NM_152678.1 | FAM116A | family with sequence similarity 116, member A | unknown | other |
| AB011146 | FAM189A1 | family with sequence similarity 189, member A1 | unknown | other |
| NM_138805.2 | FAM3D | family with sequence similarity 3, member D | Extracellular Space | cytokine |
| NM_016623.3 | FAM49B | family with sequence similarity 49, member B | unknown | other |
| NM_016605.1 | FAM53C | family with sequence similarity 53, member C | unknown | other |
| NM_152315.1 | FAM55A | family with sequence similarity 55, member A | unknown | other |
| NM_015864.2 | FAM65B | family with sequence similarity 65, member B | unknown | other |
| NM_024735.2 | FBXO31 | F-box protein 31 | unknown | other |
| NM_173558.2 | FGD2 | FYVE, RhoGEF and PH domain containing 2 | Cytoplasm | other |
| NM_173558.2 | FGD2 | FYVE, RhoGEF and PH domain containing 2 | Cytoplasm | other |
| NM_005247.2 | FGF3 | fibroblast growth factor 3 | Extracellular Space | growth factor |
| NM_007045.2 | FGFR1OP | FGFR1 oncogene partner | Cytoplasm | other |
| NM_001007529.1 | FLJ40194 | uncharacterized FLJ40194 | unknown | other |
| NM_207473.1 | FLJ41733 | FLJ41733 protein | unknown | other |
| NM_207461.1 | FLJ44881 | FLJ44881 | unknown | other |
| NM_207426.1 | FOXI2 | forkhead box I2 | Nucleus | transcription regulator |
| NM_207482.1 | FSIP2 | fibrous sheath interacting protein 2 | Cytoplasm | other |
| NM_002043.1 | GABRR2 | gamma-aminobutyric acid (GABA) A receptor, rho 2 | Plasma Membrane | ion channel |
| NM_024637.3 | GAL3ST4 | galactose-3-O-sulfotransferase 4 | Cytoplasm | enzyme |
| NM_003643.2 | GCM1 | glial cells missing homolog 1 (Drosophila) | Nucleus | transcription regulator |
| AK091091 | GDF11 | growth differentiation factor 11 | Extracellular Space | growth factor |
| NM_005268.2 | GJB5 | gap junction protein, beta 5, 31.1kDa | Plasma Membrane | transporter |
| NM_000171.1 | GLRA1 | glycine receptor, alpha 1 | Plasma Membrane | ion channel |
| NM_004877.1 | GMFG | glia maturation factor, gamma | Cytoplasm | growth factor |
| BM994423 | GMPR | guanosine monophosphate reductase | Cytoplasm | enzyme |
| NM_145171.2 | GPHB5 | glycoprotein hormone beta 5 | Extracellular Space | other |
| NM_005278.3 | GPM6B | glycoprotein M6B | Plasma Membrane | other |
| NM_181791.1 | GPR141 | G protein-coupled receptor 141 | Plasma Membrane | G-protein coupled receptor |
| NM_000273.1 | GPR143 | G protein-coupled receptor 143 | Plasma Membrane | G-protein coupled receptor |
| NM_018970.3 | GPR85 | G protein-coupled receptor 85 | Plasma Membrane | G-protein coupled receptor |
| NM_001509.1 | GPX5 | glutathione peroxidase 5 (epididymal androgen-related protein) | Extracellular Space | enzyme |
| AL133661 | GRAMD1C | GRAM domain containing 1C | unknown | other |
| NM_152407.2 | GRPEL2 | GrpE-like 2, mitochondrial (E. coli) | Cytoplasm | other |
| NM_178171.2 | GSDMA | gasdermin A | Cytoplasm | other |
| NM_003642.1 | HAT1 | histone acetyltransferase 1 | Nucleus | enzyme |
| NR_002139.1 | HCG4 | HLA complex group 4 (non-protein coding) | unknown | other |
| NM_139205.1 | HDAC5 | histone deacetylase 5 | Nucleus | transcription regulator |
| NM_014799.2 | HEPH | hephaestin | Plasma Membrane | transporter |
| NM_018194.1 | HHAT | hedgehog acyltransferase | Cytoplasm | enzyme |
| NM_021066.2 | HIST1H2AJ | histone cluster 1, H2aj | Nucleus | other |
| CA310244 | HIST1H2BN | histone cluster 1, H2bn | Nucleus | other |
| NM_003531.2 | HIST1H3A (includes others) | histone cluster 1, H3a | Nucleus | other |
| CF272033 | HIST1H3A (includes others) | histone cluster 1, H3a | Nucleus | other |
| NM_003518.3 | HIST2H2BE (includes others) | histone cluster 2, H2be | Nucleus | other |
| NM_006734.2 | HIVEP2 | human immunodeficiency virus type I enhancer binding protein 2 | Nucleus | transcription regulator |
| NM_002134.2 | HMOX2 | heme oxygenase (decycling) 2 | Cytoplasm | enzyme |
| AK093987 | HOTTIP | HOXA distal transcript antisense RNA (non-protein coding) | unknown | other |
| BC047481 | HOXD-AS1 | HOXD cluster antisense RNA 1 (non-protein coding) | unknown | other |
| NM_178135.2 | HSD17B13 | hydroxysteroid (17-beta) dehydrogenase 13 | Extracellular Space | enzyme |
| BU517060 | HSPE1 | heat shock 10kDa protein 1 (chaperonin 10) | Cytoplasm | enzyme |
| NM_130770.2 | HTR3C | 5-hydroxytryptamine (serotonin) receptor 3C, ionotropic | Plasma Membrane | ion channel |
| NM_182589.2 | HTR3E | 5-hydroxytryptamine (serotonin) receptor 3E, ionotropic | Plasma Membrane | transmembrane receptor |
| NM_053044.2 | HTRA3 | HtrA serine peptidase 3 | Extracellular Space | peptidase |
| NM_020962.1 | IGDCC4 | immunoglobulin superfamily, DCC subclass, member 4 | Plasma Membrane | other |
| NM_002180.1 | IGHMBP2 | immunoglobulin mu binding protein 2 | Nucleus | enzyme |
| NM_003639.2 | IKBKG | inhibitor of kappa light polypeptide gene enhancer in B-cells, kinase gamma | Nucleus | kinase |
| NM_175061.2 | JAZF1 | JAZF zinc finger 1 | Nucleus | transcription regulator |
| NM_002237.2 | KCNG1 | potassium voltage-gated channel, subfamily G, member 1 | Plasma Membrane | ion channel |
| NM_033272.2 | KCNH7 | potassium voltage-gated channel, subfamily H (eag-related), member 7 | Plasma Membrane | ion channel |
| NM_002251.3 | KCNS1 | potassium voltage-gated channel, delayed-rectifier, subfamily S, member 1 | Plasma Membrane | ion channel |
| NM_023930.2 | KCTD14 | potassium channel tetramerisation domain containing 14 | unknown | ion channel |
| NM_014734.2 | KIAA0247 | KIAA0247 | unknown | other |
| XM_371706.3 | KIAA1109 | KIAA1109 | unknown | other |
| XM_166132.5 | KIAA1462 | KIAA1462 | unknown | other |
| NM_031217.2 | KIF18A | kinesin family member 18A | Cytoplasm | enzyme |
| NM_002258.1 | KLRB1 | killer cell lectin-like receptor subfamily B, member 1 | Plasma Membrane | transmembrane receptor |
| NM_002274.2 | KRT13 | keratin 13 | Cytoplasm | other |
| NM_181607.1 | KRTAP19-1 | keratin associated protein 19-1 | unknown | other |
| AJ296345 | KRTAP2-4 (includes others) | keratin associated protein 2-4 | unknown | other |
| NM_018490.1 | LGR4 | leucine-rich repeat containing G protein-coupled receptor 4 | Plasma Membrane | G-protein coupled receptor |
| NM_182564.1 | LINC00469 | long intergenic non-protein coding RNA 469 | unknown | other |
| AK092078 | LINC00491 | long intergenic non-protein coding RNA 491 | unknown | other |
| NM_004140.2 | LLGL1 | lethal giant larvae homolog 1 (Drosophila) | Cytoplasm | other |
| AK092497 | LOC100134259 | uncharacterized LOC100134259 | unknown | other |
| AK024897 | LOC100289255 | uncharacterized LOC100289255 | unknown | other |
| AK055877 | LOC100506328 | uncharacterized LOC100506328 | unknown | other |
| AK056728 | LOC100506655 | uncharacterized LOC100506655 | unknown | other |
| BE551664 | LOC100507629 | uncharacterized LOC100507629 | unknown | other |
| AK055023 | LOC219690 | uncharacterized LOC219690 | unknown | other |
| AK094730 | LOC283454 | uncharacterized LOC283454 | unknown | other |
| NM_201565.1 | LOC284861 | uncharacterized LOC284861 | unknown | other |
| AK095450 | LOC285540 | uncharacterized LOC285540 | unknown | other |
| NM_182600.1 | LOC286359 | uncharacterized LOC286359 | unknown | other |
| AK057135 | LOC386597 | uncharacterized LOC386597 | unknown | other |
| NM_013437.2 | LRP12 | low density lipoprotein receptor-related protein 12 | Plasma Membrane | transmembrane receptor |
| NM_005583.3 | LYL1 | lymphoblastic leukemia derived sequence 1 | Nucleus | transcription regulator |
| NM_182573.1 | LYPD5 | LY6/PLAUR domain containing 5 | unknown | other |
| NM_194317.2 | LYPD6 | LY6/PLAUR domain containing 6 | Extracellular Space | other |
| NM_130760.1 | MADCAM1 | mucosal vascular addressin cell adhesion molecule 1 | Plasma Membrane | other |
| NM_002363.3 | MAGEB1 | melanoma antigen family B, 1 | Nucleus | other |
| NM_138702.1 | MAGEC2/MAGEC3 | melanoma antigen family C, 2 | Plasma Membrane | other |
| NM_002756.2 | MAP2K3 | mitogen-activated protein kinase kinase 3 | Cytoplasm | kinase |
| NM_002376.4 | MARK3 | MAP/microtubule affinity-regulating kinase 3 | Cytoplasm | kinase |
| NM_018834.3 | MATR3 | matrin 3 | Nucleus | other |
| XR_000273.1 | MCART3P | mitochondrial carrier triple repeat 3 pseudogene | unknown | other |
| NM_030973.2 | MED25 (includes EG:292889) | mediator complex subunit 25 | Nucleus | other |
| NM_032718.2 | MFSD9 | major facilitator superfamily domain containing 9 | unknown | transporter |
| NM_005932.1 | MIPEP | mitochondrial intermediate peptidase | Cytoplasm | peptidase |
| NM_030814.4 | MIR600HG | MIR600 host gene (non-protein coding) | unknown | other |
| NM_000248.2 | MITF | microphthalmia-associated transcription factor | Nucleus | transcription regulator |
| NM_019556.1 | MOSPD1 | motile sperm domain containing 1 | unknown | other |
| NM_033066.1 | MPP4 | membrane protein, palmitoylated 4 (MAGUK p55 subfamily member 4) | Cytoplasm | kinase |
| NM_080794.2 | MRPL39 (includes EG:27393) | mitochondrial ribosomal protein L39 | Cytoplasm | other |
| NM_006069.2 | MRVI1 | murine retrovirus integration site 1 homolog | Cytoplasm | other |
| NM_006745.2 | MSMO1 | methylsterol monooxygenase 1 | Cytoplasm | enzyme |
| NM_006636.2 | MTHFD2 | methylenetetrahydrofolate dehydrogenase (NADP+ dependent) 2, methenyltetrahydrofolate cyclohydrolase | Cytoplasm | enzyme |
| NM_019041.3 | MTRF1L | mitochondrial translational release factor 1-like | Cytoplasm | translation regulator |
| NM_002477.1 | MYL5 | myosin, light chain 5, regulatory | Cytoplasm | other |
| XM_495961.1 | MZT1 | mitotic spindle organizing protein 1 | Cytoplasm | other |
| BM907775 | NCAPD2 | non-SMC condensin I complex, subunit D2 | Nucleus | other |
| NM_021076.2 | NEFH | neurofilament, heavy polypeptide | Cytoplasm | other |
| NM_006164.2 | NFE2L2 | nuclear factor (erythroid-derived 2)-like 2 | Nucleus | transcription regulator |
| NM_024522.1 | NKAIN1 | Na+/K+ transporting ATPase interacting 1 | unknown | other |
| AI795953 | NRG2 | neuregulin 2 | Extracellular Space | growth factor |
| NM_018159.2 | NUDT11 | nudix (nucleoside diphosphate linked moiety X)-type motif 11 | Cytoplasm | phosphatase |
| NM_181745.2 | O3FAR1 | omega-3 fatty acid receptor 1 | Plasma Membrane | G-protein coupled receptor |
| NM_006189.1 | OMP | olfactory marker protein | Cytoplasm | other |
| NM_173591.1 | OTOGL | otogelin-like | unknown | other |
| NM_000915.2 | OXT | oxytocin, prepropeptide | Extracellular Space | other |
| NM_023914.2 | P2RY13 | purinergic receptor P2Y, G-protein coupled, 13 | Plasma Membrane | G-protein coupled receptor |
| NM_178129.3 | P2RY8 | purinergic receptor P2Y, G-protein coupled, 8 | Plasma Membrane | G-protein coupled receptor |
| NM_018216.1 | PANK4 | pantothenate kinase 4 | Cytoplasm | kinase |
| NM_152268.2 | PARS2 | prolyl-tRNA synthetase 2, mitochondrial (putative) | Cytoplasm | enzyme |
| NM_019119.3 | PCDHB9 | protocadherin beta 9 | Plasma Membrane | other |
| NM_002861.1 | PCYT2 | phosphate cytidylyltransferase 2, ethanolamine | Cytoplasm | enzyme |
| NM_000284.1 | PDHA1 | pyruvate dehydrogenase (lipoamide) alpha 1 | Cytoplasm | enzyme |
| NM_018444.2 | PDP1 | pyruvate dehyrogenase phosphatase catalytic subunit 1 | Cytoplasm | phosphatase |
| NM_004567.2 | PFKFB4 | 6-phosphofructo-2-kinase/fructose-2,6-biphosphatase 4 | Cytoplasm | kinase |
| NM_018288.2 | PHF10 | PHD finger protein 10 | Nucleus | other |
| NM_017934.4 | PHIP | pleckstrin homology domain interacting protein | Nucleus | other |
| NM_020901.1 | PHRF1 | PHD and ring finger domains 1 | Nucleus | other |
| AK126860 | PI4KAP2 | phosphatidylinositol 4-kinase, catalytic, alpha pseudogene 2 | unknown | other |
| NM_006221.1 | PIN1 | peptidylprolyl cis/trans isomerase, NIMA-interacting 1 | Nucleus | enzyme |
| NM_015993.1 | PLLP | plasmolipin | Plasma Membrane | transporter |
| NM_020360.2 | PLSCR3 | phospholipid scramblase 3 | Cytoplasm | enzyme |
| NM_031887.2 | PMCHL1 | pro-melanin-concentrating hormone-like 1, pseudogene | Extracellular Space | other |
| NM_004279.1 | PMPCB | peptidase (mitochondrial processing) beta | Cytoplasm | peptidase |
| NM_006468.5 | POLR3C | polymerase (RNA) III (DNA directed) polypeptide C (62kD) | Nucleus | enzyme |
| NM_176895.1 | PPAP2A | phosphatidic acid phosphatase type 2A | Plasma Membrane | phosphatase |
| NM_139126.2 | PPIL4 | peptidylprolyl isomerase (cyclophilin)-like 4 | Nucleus | enzyme |
| NM_178494.2 | PPM1N | protein phosphatase, Mg2+/Mn2+ dependent, 1N (putative) | unknown | other |
| AK128559 | PPP2R3C | protein phosphatase 2, regulatory subunit B'', gamma | Cytoplasm | other |
| NM_032319.1 | PRADC1 | protease-associated domain containing 1 | Extracellular Space | other |
| NM_052996.2 | PRDM7 | PR domain containing 7 | Nucleus | other |
| NM_198859.1 | PRICKLE2 | prickle homolog 2 (Drosophila) | Nucleus | other |
| BU854638 | PRM1 (includes EG:19118) | protamine 1 | Nucleus | other |
| BX107899 | PTCHD1 | patched domain containing 1 | Plasma Membrane | other |
| NM_002820.2 | PTHLH | parathyroid hormone-like hormone | Extracellular Space | other |
| NM_032781.2 | PTPN5 | protein tyrosine phosphatase, non-receptor type 5 (striatum-enriched) | Cytoplasm | phosphatase |
| NM_005609.1 | PYGM | phosphorylase, glycogen, muscle | Cytoplasm | enzyme |
| NM_014925.2 | R3HDM2 | R3H domain containing 2 | Nucleus | other |
| NM_030981.1 | RAB1B | RAB1B, member RAS oncogene family | Cytoplasm | enzyme |
| NM_002884.1 | RAP1A | RAP1A, member of RAS oncogene family | Cytoplasm | enzyme |
| NM_024805.1 | RBFA | ribosome binding factor A (putative) | Cytoplasm | other |
| NM_006507.2 | REG1B | regenerating islet-derived 1 beta | Extracellular Space | other |
| XM_376830.2 | RGP1 (includes EG:242406) | RGP1 retrograde golgi transport homolog (S. cerevisiae) | unknown | other |
| NM_015653.2 | RIBC2 | RIB43A domain with coiled-coils 2 | unknown | other |
| NM_024539.3 | RNF128 | ring finger protein 128, E3 ubiquitin protein ligase | Cytoplasm | enzyme |
| NM_173647.2 | RNF149 | ring finger protein 149 | unknown | other |
| XM_290732 | RNF157 | ring finger protein 157 | unknown | other |
| BF303909 | RPS3 | ribosomal protein S3 | Cytoplasm | enzyme |
| BF510484 | RQCD1 | RCD1 required for cell differentiation1 homolog (S. pombe) | Cytoplasm | other |
| NM_025158.2 | RUFY1 | RUN and FYVE domain containing 1 | Cytoplasm | transporter |
| NM_152552.1 | SAMD3 | sterile alpha motif domain containing 3 | unknown | other |
| BU607563 | SCGB3A1 | secretoglobin, family 3A, member 1 | Extracellular Space | cytokine |
| NM_054023.2 | SCGB3A2 | secretoglobin, family 3A, member 2 | Extracellular Space | other |
| NM_198081.1 | SCML4 | sex comb on midleg-like 4 (Drosophila) | unknown | other |
| NM_006922.2 | SCN3A | sodium channel, voltage-gated, type III, alpha subunit | Plasma Membrane | ion channel |
| NM_003901.2 | SGPL1 | sphingosine-1-phosphate lyase 1 | Cytoplasm | enzyme |
| NM_003026.1 | SH3GL2 | SH3-domain GRB2-like 2 | Plasma Membrane | enzyme |
| NM_003951.2 | SLC25A14 | solute carrier family 25 (mitochondrial carrier, brain), member 14 | Cytoplasm | transporter |
| NM_024698.4 | SLC25A22 | solute carrier family 25 (mitochondrial carrier: glutamate), member 22 | Cytoplasm | transporter |
| NM_152264.2 | SLC39A13 | solute carrier family 39 (zinc transporter), member 13 | Cytoplasm | transporter |
| NM_033409.2 | SLC52A3 | solute carrier family 52, riboflavin transporter, member 3 | Plasma Membrane | other |
| BM980416 | SLC5A1 | solute carrier family 5 (sodium/glucose cotransporter), member 1 | Plasma Membrane | transporter |
| NM_178498.2 | SLC5A12 | solute carrier family 5 (sodium/glucose cotransporter), member 12 | unknown | transporter |
| BG570212 | SLC6A4 | solute carrier family 6 (neurotransmitter transporter, serotonin), member 4 | Plasma Membrane | transporter |
| NM_014270.3 | SLC7A9 | solute carrier family 7 (glycoprotein-associated amino acid transporter light chain, bo,+ system), member 9 | Plasma Membrane | transporter |
| NM_005445.2 | SMC3 | structural maintenance of chromosomes 3 | Nucleus | other |
| NM_012437.3 | SNAPIN | SNAP-associated protein | Cytoplasm | other |
| NM_003096.2 | SNRPG | small nuclear ribonucleoprotein polypeptide G | Nucleus | other |
| NM_006461.2 | SPAG5 | sperm associated antigen 5 | Nucleus | peptidase |
| NM_012443.2 | SPAG6 | sperm associated antigen 6 | Cytoplasm | other |
| NM_174927.1 | SPATA19 | spermatogenesis associated 19 | unknown | other |
| NM_030965.1 | ST6GALNAC5 | ST6 (alpha-N-acetyl-neuraminyl-2,3-beta-galactosyl-1,3)-N-acetylgalactosaminide alpha-2,6-sialyltransferase 5 | Cytoplasm | enzyme |
| NM_003898.2 | SYNJ2 | synaptojanin 2 | Cytoplasm | phosphatase |
| NM_001059.1 | TACR3 | tachykinin receptor 3 | Plasma Membrane | G-protein coupled receptor |
| NM_013351.1 | TBX21 | T-box 21 | Nucleus | transcription regulator |
| NM_018426.1 | TMEM63B | transmembrane protein 63B | unknown | other |
| NM_032824.1 | TMEM87B | transmembrane protein 87B | unknown | other |
| NM_002160.1 | TNC (includes EG:116640) | tenascin C | Extracellular Space | other |
| NM_000363.3 | TNNI3 | troponin I type 3 (cardiac) | Cytoplasm | transporter |
| BG473946 | TOMM7 | translocase of outer mitochondrial membrane 7 homolog (yeast) | Cytoplasm | transporter |
| NM_000546.2 | TP53 (includes EG:22059) | tumor protein p53 | Nucleus | transcription regulator |
| NM_004179.1 | TPH1 | tryptophan hydroxylase 1 | Cytoplasm | enzyme |
| NM_003292.1 | TPR | translocated promoter region, nuclear basket protein | Nucleus | other |
| NM_144725.2 | TTC23L | tetratricopeptide repeat domain 23-like | unknown | other |
| XM_027236.5 | TTC9 | tetratricopeptide repeat domain 9 | unknown | other |
| NM_014657.1 | TTI1 (includes EG:41189) | TELO2 interacting protein 1 | unknown | other |
| NR_001549.1 | TTTY19 | testis-specific transcript, Y-linked 19 (non-protein coding) | unknown | other |
| NR_001525.1 | TTTY4 | testis-specific transcript, Y-linked 4 (non-protein coding) | unknown | other |
| NM_003321.3 | TUFM | Tu translation elongation factor, mitochondrial | Cytoplasm | translation regulator |
| NM_020648.3 | TWSG1 | twisted gastrulation homolog 1 (Drosophila) | Extracellular Space | other |
| BQ056428 | TYMS | thymidylate synthetase | Nucleus | enzyme |
| AK097488 | UBAC2-AS1 | UBAC2 antisense RNA 1 (non-protein coding) | unknown | other |
| NM_003340.4 | UBE2D3 | ubiquitin-conjugating enzyme E2D 3 | unknown | enzyme |
| NM_013319.1 | UBIAD1 | UbiA prenyltransferase domain containing 1 | Nucleus | enzyme |
| CD358078 | ULK4P2 | unc-51-like kinase 4 (C. elegans) pseudogene 2 | unknown | other |
| NM_173568.2 | UMODL1 | uromodulin-like 1 | Cytoplasm | other |
| NM_201402.1 | USP17L2 (includes others) | ubiquitin specific peptidase 17-like 2 | unknown | peptidase |
| NM_024621.1 | VEPH1 | ventricular zone expressed PH domain homolog 1 (zebrafish) | Nucleus | other |
| BM455138 | WAS | Wiskott-Aldrich syndrome (eczema-thrombocytopenia) | Cytoplasm | other |
| AL833550 | XPO1 | exportin 1 (CRM1 homolog, yeast) | Nucleus | transporter |
| NM_012255.3 | XRN2 | 5'-3' exoribonuclease 2 | Nucleus | enzyme |
| NM_144604.2 | ZC3H18 | zinc finger CCCH-type containing 18 | Nucleus | other |
| XM_372124.2 | ZCCHC6 | zinc finger, CCHC domain containing 6 | unknown | enzyme |
| NM_032226.1 | ZCCHC7 | zinc finger, CCHC domain containing 7 | Nucleus | other |
| BX640748 | ZFHX3 | zinc finger homeobox 3 | Nucleus | transcription regulator |
| NM_152283.1 | ZFP62 | zinc finger protein 62 homolog (mouse) | Nucleus | other |
| NM_007222.2 | ZHX1 | zinc fingers and homeoboxes 1 | Nucleus | transcription regulator |
| XM_048070.4 | ZNF292 | zinc finger protein 292 | Nucleus | transcription regulator |
| NM_194325.1 | ZNF30 | zinc finger protein 30 | Nucleus | other |
| NM_014594.1 | ZNF354C | zinc finger protein 354C | Nucleus | other |
| NM_015871.2 | ZNF593 | zinc finger protein 593 | Nucleus | transcription regulator |
| NM_145233.2 | ZNF625 | zinc finger protein 625 | unknown | other |
| NM_203374.1 | ZNF784 | zinc finger protein 784 | unknown | other |
| NM_007135.1 | ZNF79 | zinc finger protein 79 | Nucleus | other |
| BF511259 | unknown | unknown | unknown | unknown |
| NM_032710.1 | unknown | unknown | unknown | unknown |
| AW451323 | unknown | unknown | unknown | unknown |
| AW968163 | unknown | unknown | unknown | unknown |
| BX107948 | unknown | unknown | unknown | unknown |
| AJ318805 | unknown | unknown | unknown | unknown |
| XM_495900.1 | unknown | unknown | unknown | unknown |
| NA | unknown | unknown | unknown | unknown |
| NA | unknown | unknown | unknown | unknown |
| XM_371586.3 | unknown | unknown | unknown | unknown |
| NA | unknown | unknown | unknown | unknown |
| AW578902 | unknown | unknown | unknown | unknown |
| NA | unknown | unknown | unknown | unknown |
| NA | unknown | unknown | unknown | unknown |
| BX367797 | unknown | unknown | unknown | unknown |
| XM_097351.3 | unknown | unknown | unknown | unknown |
| AI204439 | unknown | unknown | unknown | unknown |
| NA | unknown | unknown | unknown | unknown |
| BQ771693 | unknown | unknown | unknown | unknown |
| NA | unknown | unknown | unknown | unknown |
| XM_497855.1 | unknown | unknown | unknown | unknown |
| NA | unknown | unknown | unknown | unknown |
| XM_496241.1 | unknown | unknown | unknown | unknown |
| NA | unknown | unknown | unknown | unknown |
| XM_499576.1 | unknown | unknown | unknown | unknown |
| NA | unknown | unknown | unknown | unknown |
| NA | unknown | unknown | unknown | unknown |
| NA | unknown | unknown | unknown | unknown |
| BQ028149 | unknown | unknown | unknown | unknown |
| BG213511 | unknown | unknown | unknown | unknown |
| NA | unknown | unknown | unknown | unknown |
| NM_147131.1 | unknown | unknown | unknown | unknown |
| NA | unknown | unknown | unknown | unknown |
| NA | unknown | unknown | unknown | unknown |
| AK125166 | unknown | unknown | unknown | unknown |
| BF667441 | unknown | unknown | unknown | unknown |
| XM_498210.1 | unknown | unknown | unknown | unknown |
| BC045781 | unknown | unknown | unknown | unknown |
| NA | unknown | unknown | unknown | unknown |
| NA | unknown | unknown | unknown | unknown |
| NA | unknown | unknown | unknown | unknown |
| BC038580 | unknown | unknown | unknown | unknown |
| NA | unknown | unknown | unknown | unknown |
| AA448417 | unknown | unknown | unknown | unknown |
| BX091086 | unknown | unknown | unknown | unknown |
| AW971123 | unknown | unknown | unknown | unknown |
| NA | unknown | unknown | unknown | unknown |
| NA | unknown | unknown | unknown | unknown |
| BX106736 | unknown | unknown | unknown | unknown |
| XM_499124.1 | unknown | unknown | unknown | unknown |
| XM_372992.1 | unknown | unknown | unknown | unknown |
| XM_496593.1 | unknown | unknown | unknown | unknown |
| XM_378546.1 | unknown | unknown | unknown | unknown |
| NM_153691.4 | unknown | unknown | unknown | unknown |
| BU680705 | unknown | unknown | unknown | unknown |
| BC005107 | unknown | unknown | unknown | unknown |
| NA | unknown | unknown | unknown | unknown |
| NA | unknown | unknown | unknown | unknown |
| XM_372777.2 | unknown | unknown | unknown | unknown |
| XM_378985.1 | unknown | unknown | unknown | unknown |
| NM_006508.1 | unknown | unknown | unknown | unknown |
| BX504911 | unknown | unknown | unknown | unknown |
| AA680045 | unknown | unknown | unknown | unknown |
| AK026468 | unknown | unknown | unknown | unknown |
| BC037818 | unknown | unknown | unknown | unknown |
| XM_498589.1 | unknown | unknown | unknown | unknown |
| BM686937 | unknown | unknown | unknown | unknown |
| NA | unknown | unknown | unknown | unknown |
| XM_499593.1 | unknown | unknown | unknown | unknown |
| XM_373495.2 | unknown | unknown | unknown | unknown |
| CA433649 | unknown | unknown | unknown | unknown |
| AI423125 | unknown | unknown | unknown | unknown |
| NA | unknown | unknown | unknown | unknown |
| AK056868 | unknown | unknown | unknown | unknown |
| NA | unknown | unknown | unknown | unknown |
| XM_370843.3 | unknown | unknown | unknown | unknown |
| CR602569 | unknown | unknown | unknown | unknown |
| NA | unknown | unknown | unknown | unknown |
| BG403910 | unknown | unknown | unknown | unknown |
| XM_496983.1 | unknown | unknown | unknown | unknown |
| BX537738 | unknown | unknown | unknown | unknown |
| XM_373686.2 | unknown | unknown | unknown | unknown |
| BX094902 | unknown | unknown | unknown | unknown |
| XM_498596.1 | unknown | unknown | unknown | unknown |
| NA | unknown | unknown | unknown | unknown |
| BM727497 | unknown | unknown | unknown | unknown |
| AK095045 | unknown | unknown | unknown | unknown |
| AW451676 | unknown | unknown | unknown | unknown |
| NM_182570.1 | unknown | unknown | unknown | unknown |
| AK092157 | unknown | unknown | unknown | unknown |
| NA | unknown | unknown | unknown | unknown |
| XM_378453.1 | unknown | unknown | unknown | unknown |
| XM_373742.3 | unknown | unknown | unknown | unknown |
| NA | unknown | unknown | unknown | unknown |
| NA | unknown | unknown | unknown | unknown |
| BM995578 | unknown | unknown | unknown | unknown |
| BM979825 | unknown | unknown | unknown | unknown |
| NA | unknown | unknown | unknown | unknown |
| CR749478 | unknown | unknown | unknown | unknown |
| AW301941 | unknown | unknown | unknown | unknown |
| CA413744 | unknown | unknown | unknown | unknown |
| BI912738 | unknown | unknown | unknown | unknown |
| XM_208658.5 | unknown | unknown | unknown | unknown |
| BM979422 | unknown | unknown | unknown | unknown |
| CN278569 | unknown | unknown | unknown | unknown |
| NA | unknown | unknown | unknown | unknown |
| NA | unknown | unknown | unknown | unknown |
| XM_497719.1 | unknown | unknown | unknown | unknown |
| AK090811 | unknown | unknown | unknown | unknown |
| XM_378917.2 | unknown | unknown | unknown | unknown |
| XM_372609.2 | unknown | unknown | unknown | unknown |
| XM_496652.1 | unknown | unknown | unknown | unknown |
| XM_498334.1 | unknown | unknown | unknown | unknown |
| NA | unknown | unknown | unknown | unknown |
| CB164586 | unknown | unknown | unknown | unknown |
| BX090855 | unknown | unknown | unknown | unknown |
| NA | unknown | unknown | unknown | unknown |
| NA | unknown | unknown | unknown | unknown |
| XM_373821.3 | unknown | unknown | unknown | unknown |
| NA | unknown | unknown | unknown | unknown |
| XM_499465.1 | unknown | unknown | unknown | unknown |
| BX647249 | unknown | unknown | unknown | unknown |
| XM_372125.2 | unknown | unknown | unknown | unknown |
| AF351612 | unknown | unknown | unknown | unknown |
| AK023831 | unknown | unknown | unknown | unknown |
| XM_374059.1 | unknown | unknown | unknown | unknown |
| NA | unknown | unknown | unknown | unknown |
| NA | unknown | unknown | unknown | unknown |
| XM_379774.2 | unknown | unknown | unknown | unknown |
| AK130218 | unknown | unknown | unknown | unknown |
| BM717102 | unknown | unknown | unknown | unknown |
| XM_379318.2 | unknown | unknown | unknown | unknown |
| XM_373871.2 | unknown | unknown | unknown | unknown |
| BM688644 | unknown | unknown | unknown | unknown |
| XM_374138.2 | unknown | unknown | unknown | unknown |
| BC015458 | unknown | unknown | unknown | unknown |
| XM_371111.3 | unknown | unknown | unknown | unknown |
