# Supplementary material for: Gene expression modifications in Wharton’s Jelly mesenchymal stem cells promoted by prolonged in vitro culturing
Source: BMC Genomics. 2013 Sep 21;14:635. doi: 10.1186/1471-2164-14-635 (PMC3849041; doi:10.1186/1471-2164-14-635)
Supplement: Additional file 6 — Western Blot Method. [file 1471-2164-14-635-S6.docx]

**Supplemental Materials and Methods**

**Antibodies**

Anti-Histone H3 (FL-136), anti-p53 (DO-1) and anti IL-1β (H153) antibodies were obtained from Santa Cruz Biotechnology. Secondary appropriate Horse Radish Peroxidase (HRP)-conjugated secondary antibodies were obtained from Calbiochem.

**Wester blotting**

A total of 1 × 10^6^ cells were harvested by centrifugation and solubilized in a lysis buffer containing 50 mM Tris-HCl (pH 7.4), 150 mM NaCl, 1% IGEPAL CA-630 (Sigma-Aldrich), 0.1 % sodium dodecyl sulfate (Sigma-Aldrich), 0.5 % deoxycholic acid sodium salt added with 1 mM Na2VO4, 1 μg/ml leupeptin, 1 μg/ml aprotinin and 0.5 mM phenyl-methane-sulfonyl-fluoride. Total lysates (10 μg of protein) were separated on NuPAGE 4-12% Bis-Tris Gel (Invitrogen) and blotted onto nitrocellulose membrane (Amersham). After saturation with 5% skim milk powder (Sigma-Aldrich) in tris buffer saline pH 7.4 and 0.05 % tween20 (Sigma-Aldrich), the membranes were incubated with 0.2 μg/ml anti-HIST1H3C, anti-P53, anti-IL1β and anti-β-actin (internal control) antibodies followed by the appropriate HRP-conjugated secondary antibodies (0.27 μg/ml). Immunoreactive bands were detected by using the ECL system (Santa Cruz Biotechnology), according to the manufacturer’s instructions. Chemiluminescence was detected by Gel Doc (Bio Rad) and analyzed by Quantity ONE 4.3.1 (Bio Rad) (78)
